# Life course health consequences and associated annual costs of adverse childhood experiences across Europe and North America: a systematic review and meta-analysis

**DOI:** 10.1016/S2468-2667(19)30145-8

**Published:** 2019-09-03

**Authors:** Mark A Bellis, Karen Hughes, Kat Ford, Gabriela Ramos Rodriguez, Dinesh Sethi, Jonathon Passmore

**Affiliations:** a Policy and International Health Directorate, World Health Organization Collaborating Centre on Investment for Health and Wellbeing, Public Health Wales, Wrexham, UK; b College of Human Sciences, Bangor University, Wrexham, UK; c Violence and Injury Prevention, World Health Organization Regional Office for Europe, Copenhagen, Denmark

## Abstract

**Background:**

An increasing number of studies are identifying associations between adverse childhood experiences (ACEs) and ill health throughout the life course. We aimed to calculate the proportions of major risk factors for and causes of ill health that are attributable to one or multiple types of ACE and the associated financial costs.

**Methods:**

In this systematic review and meta-analysis, we searched for studies in which risk data in individuals with ACEs were compared with these data in those without ACEs. We searched six electronic databases (MEDLINE, CINAHL, PsycINFO, Applied Social Sciences Index and Abstracts, Criminal Justice Databases, and the Education Resources Information Center) for quantitative studies published between Jan 1, 1990, and July 11, 2018, that reported risks of health-related behaviours and causes of ill health in adults that were associated with cumulative measures of ACEs (ie, number of ACEs). We included studies in adults in populations that did not have a high risk of ACEs, that had sample sizes of at least 1000 people, and that provided ACE prevalence data. We calculated the pooled RR for risk factors (harmful alcohol use, illicit drug use, smoking, and obesity) and causes of ill health (cancer, diabetes, cardiovascular disease, respiratory disease, anxiety, and depression) associated with ACEs. RRs were used to estimate the population-attributable fractions (PAFs) of risk attributable to ACEs and the disability-adjusted life-years (DALYs) and financial costs associated with ACEs. This study was prospectively registered in PROSPERO (CRD42018090356).

**Findings:**

Of 4387 unique articles found following our initial search, after review of the titles (and abstracts, when the title was relevant), we assessed 880 (20%) full-text articles. We considered 221 (25%) full-text articles for inclusion, of which 23 (10%) articles met all selection criteria for our meta-analysis. We found a pooled prevalence of 23·5% of individuals (95% CI 18·7–28·5) with one ACE and 18·7% (14·7–23·2) with two or more ACEs in Europe (from ten studies) and of 23·4% of individuals (22·0–24·8) with one ACE and 35·0% (31·6–38·4) with two or more ACEs in north America (from nine studies). Illicit drug use had the highest PAFs associated with ACEs of all the risk factors assessed in both regions (34·1% in Europe; 41·1% in north America). In both regions, PAFs of causes of ill health were highest for mental illness outcomes: ACEs were attributed to about 30% of cases of anxiety and 40% of cases of depression in north America and more than a quarter of both conditions in Europe. Costs of cardiovascular disease attributable to ACEs were substantially higher than for most other causes of ill health because of higher DALYs for this condition. Total annual costs attributable to ACEs were estimated to be US$581 billion in Europe and $748 billion in north America. More than 75% of these costs arose in individuals with two or more ACEs.

**Interpretation:**

Millions of adults across Europe and north America live with a legacy of ACEs. Our findings suggest that a 10% reduction in ACE prevalence could equate to annual savings of 3 million DALYs or $105 billion. Programmes to prevent ACEs and moderate their effects are available. Rebalancing expenditure towards ensuring safe and nurturing childhoods would be economically beneficial and relieve pressures on health-care systems.

**Funding:**

World Health Organization Regional Office for Europe.

## Introduction

An increasing number of studies^1^ have identified the long-term effects of adverse childhood experiences (ACEs) on health throughout the life course. The term ACEs refers to some of the most intense sources of stress that children can be exposed to, including child maltreatment, interparental violence, and parental substance use. Along with immediate health and educational effects,^2^ ACEs have been linked to higher risks of health-harming behaviours, including smoking, harmful alcohol consumption, and drug use.^1^, ^3^, ^4^ Exposure to ACEs is also associated with an increased risk of mental illness and other conditions, including cancer and cardiovascular disease.^1^, ^4^, ^5^ The effect of ACEs on mental health and adoption of health-harming behaviours is one set of mechanisms connecting ACEs to chronic ill health.^6^ However, evidence from biomedical studies^7^ suggests that ACEs also directly affect neurological, hormonal, and immunological development. Thus, ACEs are associated with increasing biomarkers for inflammation and shortened telomeres, which is consistent with direct effects of ACEs on chronic diseases such as cancer, cardiovascular disease, and respiratory disease.^8^

**Research in context**

**Evidence before this study**

An increasing number of studies are examining the relationships between exposure to adverse childhood experiences (ACEs), such as child maltreatment and exposure to domestic violence, and health outcomes across the life course. Childhood exposure to an increasing number of ACEs has an ordinal relationship with a higher prevalence of common health risk factors (such as smoking and obesity) and long-term causes of ill health (such as cancers, cardiovascular disease, and diabetes) in later life. These effects have been demonstrated primarily in high-income countries and are supported by studies of ACE-related physiological changes consistent with increased health risks. We searched six electronic databases (MEDLINE, CINAHL, PsycINFO, Applied Social Sciences Index and Abstracts, Criminal Justice Databases, and Education Resources Information Center) to identify quantitative studies that reported risks of health-related behaviours and causes of ill health in adults that were associated with cumulative measures of ACEs (ie, number of ACEs). We used the search terms “adverse childhood experience*”, “adverse childhood event*”, and “childhood adversit*”, with no language restrictions. We supplemented searches by screening reference lists of retrieved studies and through use of research networks (ie, screening online resources maintained by active ACE researchers). We searched for all relevant studies published between Jan 1, 1990, and July 11, 2018.

**Added value of this study**

With meta-analyses and population-attributable fraction methods, we pooled data on ACE-attributable risks for leading risk factors for and causes of ill health, and we converted these into annual ACE-attributable disability-adjusted life-years (DALYs) for Europe and north America. We calculated the absolute measures of ill health that could potentially be avoidable by preventing ACEs in childhoods across each global region. Finally, through a human capital approach, we expressed ACE-attributable ill health (in DALYs) as annual financial costs. We estimated that the annual costs from the effect of ACEs on the health outcomes measured were US$581 billion in Europe (equivalent to 2·67% of gross domestic product) and $748 billion in north America (equivalent to 3·55% of gross domestic product).

**Implications of all the available evidence**

Despite growing epidemiological and physiological evidence, the effect of ACEs on national and international health policy has been slow to emerge. Our findings suggest that ACEs are consistently an avoidable risk factor for some of the largest threats to public health and costs to health services across Europe and north America. Evidence-based approaches to preventing ACEs and moderating their effects are available. The implementation of these approaches at scale could provide millions more individuals with better quality childhoods and generate substantial cost savings.

Global estimates suggest approximately 1 billion children (aged 2–17 years) were victims of violence in the past year.^9^ Consequently, the UN Sustainable Development Goals include a target (16.2) to end all forms of violence against children. Additionally, health ministries in WHO member states have committed to a global plan of action, to strengthen the role of the health system within a national multisectoral response to address interpersonal violence, in particular against women and girls and against children.^10^ These commitments are supported by regional action plans.^11^ Although such policy initiatives can focus on specific topics (such as child maltreatment), ACEs routinely cluster in affected households, with maltreatment, domestic violence, and caregiver mental illness or substance abuse often affecting the same children. Globally, more than one in four people have a mental illness in their lifetime,^12^ and around one in three women who have been in a relationship have been subjected to intimate partner violence.^13^ Many incidents of such violence occur in households containing children and, although the harmful effects of these experiences on children are well established,^14^ there are no global measures of the numbers of children affected.

The effects of ACEs on health-harming behaviours or health conditions can be additive or multiplicative, resulting in substantially increased risks to those experiencing multiple types of ACEs.^1^ Despite the possible interaction of these effects, estimates of costs imposed by ACEs have, to date, focused on individual ACEs (such as child maltreatment)^15^, ^16^ rather than the combined costs of several ACEs. Research exploring such costs is needed to highlight the economic and health burden of ACEs and to make connections between often disjointed policies that tackle child maltreatment, domestic violence, and other ACEs separately. To our knowledge, there has been no attempt to systematically combine studies to examine the proportion of risk factors or causes of ill health that can be attributed to one or multiple ACEs. Global, regional, and national efforts to ensure safer childhoods, protect women, control substance use, and prevent long-term effects of ACEs, particularly on non-communicable diseases (NCDs), require such knowledge.

We therefore aimed to combine ACE studies from Europe and, separately, north America, to calculate the PAFs (ie, the proportion of adverse health outcomes in the population that are attributable to ACEs) for key health-harming behavioural risk factors (such as smoking) and causes of ill health (such as diabetes). We present PAFs associated with exposure to one or multiple ACEs, and we used the 2017 Global Burden of Disease (GBD) Study to calculate ACE-related disability-adjusted life years (DALYs) for each risk factor and cause. Finally, we used a human capital model^15^ to provide a financial estimate of the annual costs of ACEs to Europe and north America.

## Methods

### Search strategy and selection criteria

In this systematic review and meta-analysis, we searched for studies in which risk data in individuals with ACEs were compared with risk data in those without ACEs. We included studies that were predominantly focused on adults and populations that were not at a known high risk of ACEs; that had sample sizes of at least 1000 people; that provided prevalence data for the number of ACEs experienced (ACE count); and that presented the odds ratio (OR), relative risk (RR), or hazard ratio (HR) for one ACE and multiple ACEs (in any ACE count categories, permitting synthesis into categories of one and at least two ACEs). To be included, studies were required to have measured at least three types of ACE.

We searched six electronic databases (MEDLINE, CINAHL, PsycINFO, Applied Social Sciences Index and Abstracts, Criminal Justice Databases, and the Education Resources Information Center) to identify quantitative studies that reported risks of health-related behaviours and causes of ill health in adults that were associated with cumulative measures of ACEs (ie, number of ACEs). We used the search terms “adverse childhood experience*”, “adverse childhood event*”, and “childhood adversit*”, with no language restrictions. We supplemented searches by screening reference lists of retrieved studies and through use of research networks (ie, screening online resources maintained by active ACE researchers). KH did a search for all relevant studies published between Jan 1, 1990, and Dec 6, 2017. This search was updated on July 11, 2018. KH and GRR independently screened titles and abstracts, then full texts of articles that were judged to be relevant.

We included studies that reported summary estimates at population levels of relationships between cumulative ACEs and any of four risk factors (harmful alcohol use, illicit drug use, smoking, or obesity) or six causes of ill health (cancer, diabetes, cardiovascular disease, respiratory disease, anxiety, or depression).

### Data analysis

KH, KF, and GRR extracted data from included studies on country, study type, number of participants, population surveyed, and ACE and outcome measurements. We identified the outcomes and geographical coverage of studies by considering the number of studies available, outcomes measured, and the availability of data on DALYs associated with risks or causes of ill health within the GBD study. Thus, we included studies that were done in two global regions: Europe (WHO European Region) and north America (World Bank region; appendix p 1); and those that reported relationships between cumulative ACEs and any of the four risk factors or six causes of ill health.

Where more than one study reported data from the same sample and outcome, only one study was selected, which we based on a larger sample size or better suitability of data. Where several study outcomes could contribute to a single health condition, two reviewers (KH and GRR) independently identified the best match, with a third reviewer (MAB) resolving any conflicts. We did not use any specific outcome definitions, but individual study criteria are shown in the appendix (p 1).

RRs, HRs, or ORs were extracted at each available ACE count level (adjusted for demographics and socioeconomic status where available; table 1).^3^, ^4^, ^5^, ^17^, ^18^, ^19^, ^20^, ^21^, ^22^, ^23^, ^24^, ^25^, ^26^, ^27^, ^28^, ^29^, ^30^, ^31^, ^32^, ^33^, ^34^, ^35^, ^36^ All articles reported some sociodemographic adjustments and adjusted ORs were transformed into RRs by use of the equation: RR=OR/(1 − p_0_+ [p_0_ × OR]), where p_o_ is the baseline risk in the absence of ACEs.^37^ Adjusted positive and negative counts by condition and ACE category were also generated for use in the meta-analysis. Covariates from proportional hazards models were treated as RRs. For each study, all categories of RR for more than one ACE were combined to give an RR for two or more ACEs by use of a weighted mean method with weighting by proportions in each ACE category. Pooled RRs with 95% CIs for each risk factor and cause in each region were generated through random effects models. Where there was only one study, the RR was calculated directly from that study. We calculated pooled prevalence of individuals with one ACE and with two or more ACEs for each region with the Stuart–Ord method, and we calculated pooled RRs with the DerSimonian and Laird method (which we used to fit random effects). Both methods generated CIs for pooled and individual studies.

**Table 1:** Summary of included studies

	**Region**	**Country**	**Study type**	**Population**	**Sample size (n)**	**Age range (years)**	**ACEs measured (n)**	**ACE prevalence**			**Outcomes**	**Quality assessment**						
								0	1	≥2		Sampling*	Bias†	ACE‡	Response§	Decliners¶	Participants‖	Analysis**
Anda et al (2006)^17^	NAm	USA	C	HMO	17 337	≥19	8	36%	26%	38%	ABCDEF	0	1	1	1	0	1	1
Bellis et al (2014)^18^	Eur	UK	CS	General	1500	18–70	11	53%	19%	28%	ABCDJ	1	0	1	1	0	1	1
Bellis et al (2014)^19^	Eur	Various	CS	Students	10 696	18–25	10	47%	25%	28%	ABC	0	0	1	1	0	1	1
Björkenstam et al (2017)^20^	Eur	Sweden	C	General	478 141	24–28	8	69%	22%	10%	F	1	0	1	NA	NA	1	1
Cunningham et al (2014)^21^	NAm	USA	CS	General	45 561	≥18	8	37%	23%	40%	J	1	1	1	1	0	1	1
Dahl et al (2017)^22^	Eur	Denmark	C	General	978 647	19–34	9	48%	34%	18%	F	1	0	1	NA	NA	0	0
Dong et al (2004)^23^	NAm	USA	C	HMO	17 337	≥19	10	36%	26%	38%	H	0	1	1	1	1	1	1
Downey et al (2017)^24^	NAm	USA	CS	General	6361	≥18	8	46%	23%	31%	ACDFGHIJ	1	1	1	0	0	1	1
Felitti et al (1998)^25^	NAm	USA	C	HMO	8506	19–92	7	48%	25%	27%	GIJ	0	1	1	1	1	1	1
Ford et al (2011)^26^	NAm	USA	CS	General	25 809	≥18	8	41%	22%	37%	C	1	1	1	1	0	1	1
Ford et al (2016)^27^	Eur	UK	CS	General	5454	18–69	9	56%	18%	26%	ABCDGHIJ	1	1	1	1	0	1	1
Friedman et al (2015)^28^	NAm	USA	C	General	3996	30–84	27††	52%	28%	20%	DHI	1	0	0	0	0	1	0
Hughes et al (2018)^29^	Eur	UK	CS	General	2497	18–69	11	50%	19%	31%	EF	1	1	1	1	0	1	1
Kelly-Irving et al (2013)^30^	Eur	UK	C	General	6138	50	6	75%	20%	6%	G	1	0	1	1	0	1	1
McCrory et al (2015)^31^	Eur	Ireland	CS	General	6408	≥50	4	66%	26%	8%	GHIJ	1	0	1	1	1	1	1
Poole et al (2017)^32^	NAm	Canada	CS	Primary care	4006	18–92	10	31%	24%	46%	F	0	0	1	0	0	1	1
Wade et al (2016)^33^	NAm	USA	CS	General	1784	≥18	9‡‡	33%	21%	47%	CDFHIJ	0	1	1	1	0	1	1
Wainwright et al (2007)^34^	Eur	UK	C	Primary care	20 888	41–80	8	51%	30%	19%	J	0	1	0	0	0	0	1
Warne et al (2017)^35^	NAm	USA	CS	General	7594	≥18	10	48%	23%	29%	ACEF	1	0	1	0	0	1	0
Ye and Reyes-Salvail (2014)^36^	NAm	USA	CS	General	5928	≥18	8	42%	22%	36%	ACEFHIJ	1	1	1	1	0	1	0
Bellis et al (2014)^3^	Eur	UK	CS	General	3885	18–69	9	52%	23%	25%	ABC	1	1	1	1	0	1	1
Campbell et al (2016)^4^	NAm	USA	CS	General	48 526	≥18	11	45%	21%	34%	ACDFHI	1	1	1	0	0	1	1
Bellis et al (2015)^5^	Eur	UK	CS	General	3885	18–69	9	54%	23%	24%	GHIJ	1	1	1	1	0	1	1

Outcomes: A indicates harmful alcohol use, B indicates illicit drug use, C indicates smoking, D indicates obesity, E indicates anxiety, F indicates depression, G indicates cancer, H indicates cardiovascular disease, I indicates diabetes, and J indicates respiratory disease. Quality assessment: 1 indicates the study met the criteria and 0 indicates the study did not meet the criteria, or this element was not reported. ACE=Adverse childhood experience. C=cohort study. CS=cross-sectional study. Eur=Europe. HMO=health maintenance organisation. NAm=north America. NA=not applicable.

* Study used a nationally representative or whole population sample.

† Sample was not considered to have additional bias beyond that common to retrospective ACE studies.

‡ Validated or well described ACE measurement tool used.

§ Response rate of at least 50%; however, methods of calculating response rates varied between studies, so rating is based on available data.

¶ Information was provided on individuals who chose not to participate in the study.

‖ A demographic description of sample was provided.

** Analysis controlled for key demographics, including a socioeconomic measure.

†† Of all possible adverse events, only those occurring before age 18 years were included.

‡‡ Study measured an additional five ACEs, but relevant analyses were restricted to nine ACEs.

Heterogeneity between studies was measured with the *I*^2^ statistic.^38^ The risk of publication bias was evaluated by use of the Begg and Mazumdar and Egger tests and visual inspection of funnel plots where sufficient studies (more than ten)^38^ were available (appendix).

For each health outcome, we calculated population-attributable fractions (PAFs) for each ACE exposure level (ie, one ACE or at least two ACEs) by use of the equations:

$$PAF_{ACE1}=\frac{{\text{P}}_{\text{ACE}1}\times ({\text{RR}}_{\text{ACE}1}-1)}{\left({\text{P}}_{\text{ACE}0})+({\text{P}}_{\text{ACE}1}\times {\text{RR}}_{\text{ACE}1})+({\text{P}}_{\text{ACE}2+}\times {\text{RR}}_{\text{ACE}2+}\right)}$$

and

$$PAF_{ACE2+}=\frac{{\text{P}}_{\text{ACE}2+}\times ({\text{RR}}_{\text{ACE}2+}-1)}{\left({\text{P}}_{\text{ACE}0})+({\text{P}}_{\text{ACE}1}\times {\text{RR}}_{\text{ACE}1})+({\text{P}}_{\text{ACE}2+}\times {\text{RR}}_{\text{ACE}2+}\right)}$$

where RR_ACE(exposure)_ is the pooled RR associated with ACEs and P_ACE(exposure)_ the proportion of the sample exposed to a particular ACE count level.^39^ The benefits and limitations of PAF methods are discussed elsewhere.^40^

To calculate costs associated with each risk factor and cause, we adapted the human capital method.^15^, ^41^ Human capital methods assign a monetary value to loss of health, which is calculated as reduced economic productivity due to ill health and premature mortality. We identified a matched category in the GBD study dataset for each risk factor and cause (appendix). We extracted estimates of DALYs in 2017 for each region for the age categories 15–49 years, 50–69 years, and older than 70 years. Gross domestic product (GDP) and GDP per capita (2017 current US$) were extracted from World Bank data for north America (region) and Europe (extracted for each country and combined). Following the human capital method, we assume one DALY is equal to a region's GDP per capita, thus costs for each condition are calculated based on the number of DALYs × GDP per capita. PAFs were applied to total cost to estimate the regional economic value of DALYs lost for each risk factor and cause by ACE level (ie, one ACE or two or more ACEs). We also calculated the value of DALYs lost as a percentage of total regional GDP. To generate a total cost of ACEs, we removed DALYs from risk factors that also related to specified causes, to avoid counting twice (eg, we did not count the DALYs related to smoking that were also attributed to cancer).

In prespecified sensitivity analyses, we calculated DALYs and associated costs with upper and lower CIs for pooled RRs and ACE prevalence and, in another analysis, we also restricted DALYs to those for individuals aged 15–69 years. Data editing and calculations were done in Excel. We used StatsDirect (version 3.1.18) for meta-analyses. This study was prospectively registered in PROSPERO (CRD42018090356).

### Role of the funding source

The funder of the study contributed to study design and writing of the report. The funder of the study had no role in data collection, data analysis, or data interpretation. The corresponding author had full access to all the data in the study and had final responsibility for the decision to submit for publication.

## Results

**Figure 1:**
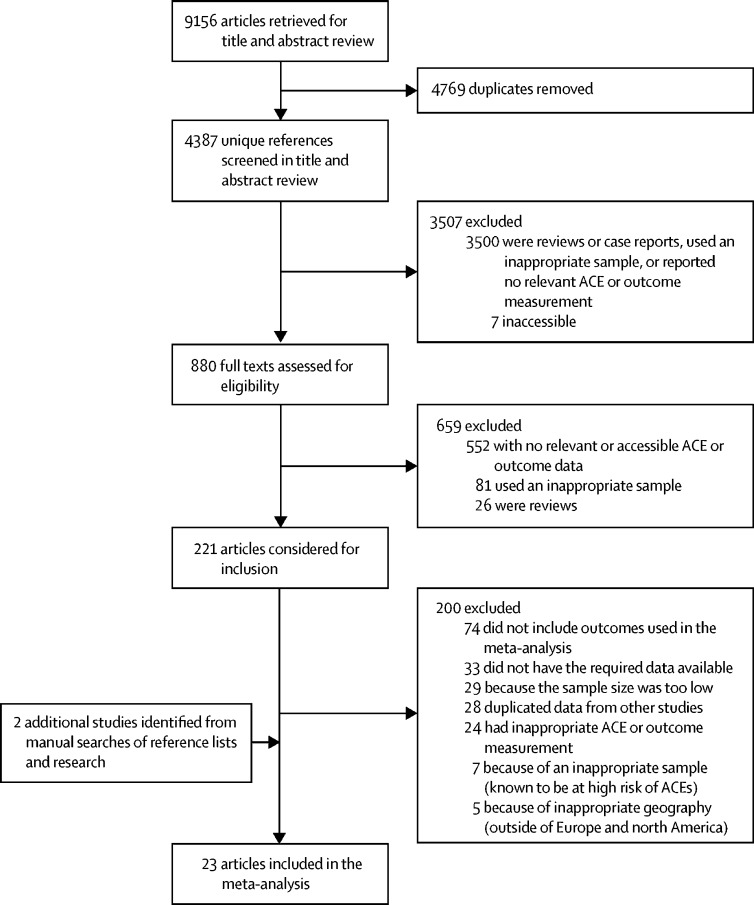
Study selection flowchart ACE=adverse childhood experience. *Did not meet study requirements.

We retrieved 9156 articles in our initial search (figure 1). After we removed duplicates (leaving 4387 [48%] unique references) and reviewed the titles (and abstracts, when the title was relevant), we assessed the 880 (10%) full texts that remained. We excluded 3507 (38%) articles after the title and abstract review, and we excluded 659 (7%) articles after full text review. We considered 221 (25%) full-text articles for inclusion. We excluded 200 (90%) of these articles because they did not include outcomes used in our meta-analysis (n=74), they did not specify the necessary data (n=33), the sample size was too low (n=29), they duplicated data from other studies (n=28), they used inappropriate methods (n=24), or they had inappropriate study populations (n=7; ie, sample was known to be at high risk of ACEs) or geography (n=5). 23 (10%) articles met all selection criteria for our meta-analysis.

The characteristics of the 23 articles in our meta-analysis are shown in table 1.^3^, ^4^, ^5^, ^17^, ^18^, ^19^, ^20^, ^21^, ^22^, ^23^, ^24^, ^25^, ^26^, ^27^, ^28^, ^29^, ^30^, ^31^, ^32^, ^33^, ^34^, ^35^, ^36^ 11 studies were based in Europe and 12 studies were in north America (predominantly the USA). Sample sizes ranged from 1500 to 978 647 people. The two largest studies^20^, ^22^ used whole-population birth cohorts. Most other studies used representative general population samples, but three studies used a health insurance sample,^17^, ^23^, ^25^ two used primary care samples,^32^, ^34^ and one used higher education samples.^19^ There was variation in number and type of ACEs measured, but most studies covered a similar core set of ACEs (appendix p 2). These ACEs included child physical, sexual, and emotional abuse; exposure to domestic violence; parental separation; and household member substance abuse, mental illness, and criminality.

Pooled RRs for risk factors and causes of ill health are shown in table 2. Forest plots of these data are shown in the appendix (p 9). Across all risk factors in both regions, pooled RRs (reference category 0 ACEs) increased more when exposed to two or more ACEs compared with one ACE. In both regions, the highest RRs were for illicit drug use (but, for north America, this RR was based on a single study), followed by harmful alcohol use, smoking, then obesity. In Europe, the lower CIs for obesity risk estimates included an RR value of 1 with one and two or more ACEs. There was considerable heterogeneity between estimates for alcohol use in both regions. Heterogeneity between estimates for smoking and obesity was also notably high in north American studies.

**Table 2:** Pooled relative risks for risk factors and causes of ill health

		Studies (n)*	**Individuals (n)**	**1 ACE**		**≥2 ACEs**	
				Pooled relative risk	Heterogeneity, *I*^2^	Pooled relative risk	Heterogeneity, *I*^2^
**Risk factors**							
Harmful alcohol use							
	Europe	4	20 427	1·51 (1·22–1·87)	75·4% (0–89·1)	2·11 (1·13–3·95)	98·0% (97·1–98·5)
	North America	5	85 745	1·44 (1·20–1·74)	80·1% (37·6–89·8)	1·81 (1·22–2·68)	96·7% (95·2–97·6)
Illicit drug use							
	Europe	4	21 365	1·69 (1·48–1·93)	45·7% (0–80·7)	2·89 (2·68–3·12)	0% (0–67·9)
	North America	1	17 337	1·53 (1·36–1·72)	NA	2·64 (2·40–2·91)	NA
Smoking							
	Europe	4	21 402	1·29 (1·21–1·38)	1% (0–68·2)	1·82 (1·71–1·95)	25·9% (0–75·5)
	North America	7	113 339	1·23 (1·04–1·46)	93·1% (88·9–95·2)	1·74 (1·47–2·05)	95·1% (92·8–96·4)
Obesity							
	Europe	2	6437	1·06 (0·99–1·15)	0%†	1·24 (0·85–1·79)	41·2%†
	North America	5	78 004	1·08 (1·00–1·17)	69·9% (0–86·2)	1·23 (1·06–1·43)	93·8% (89·1–95·9)
**Causes of ill health**							
Anxiety							
	Europe	1	2493	1·44 (1·17–1·77)	NA	2·56 (2·19–2·98)	NA
	North America	3	30 859	1·08 (0·66–1·77)	94% (85·6–96·6)	2·25 (1·43–3·56)	96·1% (92·5–97·6)
Depression							
	Europe	3	1 459 284	1·54 (1·51–1·57)	0% (0–72·9)	2·34 (2·19–2·50)	82·5% (0–92·5)
	North America	7	91 112	1·34 (1·15–1·56)	88·6% (78·5–92·8)	2·69 (2·17–3·33)	96·8% (95·6–97·5)
Cancer							
	Europe	4	21 593	1·08 (0·89–1·30)	39·6% (0–79·1)	1·58 (1·32–1·91)	25·6% (0–75·4)
	North America	2	14 372	1·10 (0·95–1·28)	0%†	1·25 (1·10–1·43)	0%†
Cardiovascular disease							
	Europe	3	15 742	1·11 (0·99–1·24)	0% (0–72·9)	1·57 (1·20–2·06)	59·9% (0–86·7)
	North America	6	83 932	1·16 (1·04–1·29)	55·2% (0–80)	1·60 (1·41–1·81)	75·7% (26·9–87·4)
Diabetes							
	Europe	3	15 733	1·02 (0·88–1·19)	0% (0–72·9)	1·43 (1·11–1·83)	58·7% (0–86·4)
	North America	6	74 662	1·11 (1·02–1·22)	39·3% (0–74·7)	1·15 (0·97–1·37)	85·3% (66·5–91·5)
Respiratory disease							
	Europe	5	38 075	1·19 (1·04–1·35)	14·7% (0–69·1)	1·98 (1·42–2·76)	83·3% (53·1–91·1)
	North America	5	67 417	1·29 (1·17–1·41)	0% (0–64·1)	1·90 (1·58–2·29)	77% (19·4–88·6)

Data in parentheses are 95% CIs. ACE=Adverse childhood experience. NA=not applicable.

* Included studies are shown in the appendix (p 9).

† CIs not calculable.

For all causes of ill health, RRs were higher with two or more ACEs than with one ACE. With one ACE, lower CIs included an RR value of 1 in both regions for cancer; in Europe, for diabetes and cardiovascular disease; and, in north America, for anxiety. The highest pooled RR associated with ACEs was reported for depression in both regions. With two or more ACEs, we found that the lowest RR was for diabetes in both regions, and the highest RR was for depression and anxiety. However, there was considerable heterogeneity between most estimates for both mental illness outcomes (where calculable). Heterogeneity between estimates was low to moderate for all physical health conditions with one ACE, and for cancer with two or more ACEs. There was substantial heterogeneity between estimates for other conditions with two or more ACEs.

We calculated regional estimates of ACE prevalence by pooling prevalence data across studies (excluding duplicate samples). Ten studies (comprising 1 514 254 individuals) in Europe provided a pooled prevalence of 23·5% of these individuals (95% CI 18·7–28·5) with one ACE and 18·7% (14·7–23·2) with two or more ACEs (appendix p 6). Nine studies (comprising 121 341 individuals) in north America provided a pooled prevalence of 23·4% (22·0–24·8) with one ACE and 35·0% (31·6–38·4) with two or more ACEs. There was high heterogeneity between estimates in both regions (appendix p 6). These regional ACE prevalence estimates were used in PAF and costs calculations.

Illicit drug use had the highest PAFs associated with ACEs of all the risk factors assessed in both regions (34·1% in Europe; 41·1% in north America, based on a single study), with estimated ACE-related annual costs of $46 billion in Europe and $168 billion in north America (table 3). Approximately a quarter of harmful alcohol use was associated with ACEs in both regions. Because of substantially higher DALYs associated with alcohol use than with drug use in Europe, the estimated ACE-attributable costs of alcohol use were more than three times higher than for drugs, reaching $143 billion (0·65% of GDP). Conversely, in north America, DALYs associated with alcohol use ($73 billion; 0·34% of GDP) were lower than those with drugs, meaning that ACE-attributable costs were also lower. ACEs were attributed to 18·2% of individuals smoking in Europe and 23·7% in north America, with estimated associated costs of $165 billion (Europe) and $160 billion (north America). Although PAFs of obesity associated with ACEs were low compared with other risk factors, ACE-attributable costs were $40 billion in Europe and $65 billion in north America.

**Table 3:** Population-attributable fractions and DALYs and costs attributable to ACEs for risk factors and causes of ill health

		**Population-attributable fractions (%)**			Total DALYs for risk factor (thousands)*	DALYs attributable to ACEs (thousands)*			**Total estimated cost of conditions (billion US$)**†	**Total attributable costs by ACE count (billion US$)**†			**Total attributable costs of ACEs (% of GDP)**
		1 ACE	≥2 ACEs	All ACEs		1 ACE	≥2 ACEs	All ACEs		1 ACE	≥2 ACEs	All ACEs	
**Risk factors**													
Harmful alcohol use													
	Europe	9·1%	15·6%	24·7%	24 478	2217	3825	6042	577	52	90	143	0·65%
	North America	7·5%	20·4%	27·9%	4474	335	915	1250	260	19	53	73	0·34%
Illicit drug use													
	Europe	10·7%	23·4%	34·1%	5702	610	1333	1944	135	14	31	46	0·21%
	North America‡	7·3%	33·8%	41·1%	7059	514	2385	2899	410	30	138	168	0·80%
Smoking													
	Europe	5·6%	12·6%	18·2%	38 541	2142	4873	7015	909	51	115	165	0·76%
	North America	4·1%	19·6%	23·7%	11 595	479	2274	2753	673	28	132	160	0·76%
Obesity													
	Europe	1·4%	4·2%	5·6%	30 072	430	1251	1681	709	10	30	40	0·18%
	North America	1·7%	7·3%	9·0%	12 542	207	917	1124	728	12	53	65	0·31%
**Causes of ill health**													
Anxiety													
	Europe‡	7·4%	20·9%	28·3%	3410	252	714	966	80	6	17	23	0·10%
	North America	1·2%	30·1%	31·3%	1986	25	597	622	115	1	35	36	0·17%
Depression													
	Europe	9·2%	18·3%	27·5%	4417	406	807	1213	104	10	19	29	0·13%
	North America	4·8%	35·3%	40·1%	1990	95	703	798	116	6	41	46	0·22%
Cancer													
	Europe	1·6%	9·7%	11·3%	43 828	716	4258	4974	1034	17	100	117	0·54%
	North America‡	2·2%	7·9%	10·1%	16 277	351	1290	1641	945	20	75	95	0·45%
Cardiovascular disease													
	Europe	2·2%	9·5%	11·7%	67 115	1492	6357	7848	1583	35	150	185	0·85%
	North America	2·9%	16·8%	19·7%	16 849	496	2825	3321	978	29	164	193	0·92%
Diabetes													
	Europe	0·5%	7·4%	7·9%	8602	41	636	677	203	1	15	16	0·07%
	North America	2·5%	5·0%	7·5%	4195	104	209	313	244	6	12	18	0·09%
Respiratory disease													
	Europe	3·6%	14·9%	18·5%	10 657	383	1591	1974	251	9	38	47	0·21%
	North America	4·8%	22·8%	27·6%	6176	298	1410	1707	359	17	82	99	0·47%

ACE=Adverse childhood experience. GDP=gross domestic product.

* Rounded to the nearest thousand.

† Rounded to the nearest billion, 2017 GDP.

‡ Based on a single study.

In both regions, PAFs of causes of ill health were highest for mental illness outcomes (table 3). ACEs were attributed to about 30% of anxiety cases and 40% of depression cases in north America and more than a quarter of both conditions in Europe. The combined annual costs of depression and anxiety attributed to ACEs were around $51 billion in Europe and $82 billion in north America. More than a quarter of cases of respiratory disease in north America and about one-fifth of cases in Europe were attributed to ACEs, with estimated costs of $99 billion (north America) and $47 billion (Europe). PAFs of cardiovascular disease and cancer to ACEs were lower than for most other causes of ill health, yet associated costs were substantially higher because of higher DALYs for these conditions. Diabetes had the lowest PAFs to ACEs of causes studied.

In both regions, most ACE-attributable costs were accounted for by exposure to two or more ACEs (*vs* one ACE; table 3). The proportions of costs accounted for by individuals with two or more ACEs ranged from 63% for alcohol use in Europe ($90 billion, of a total ACE-attributable cost of $143 billion) to 96% for anxiety in north America ($35 billion of $36 billion). Total annual costs attributable to ACEs were estimated to be $581 billion in Europe (equivalent to 2·67% of GDP) and $748 billion in north America (equivalent to 3·55% of GDP). In Europe, 77% of these costs arose in individuals with two or more ACEs whereas, in north America, this value was 82%. A 10% reduction in ACE prevalence (to values of 21·1% with one ACE and 16·9% with two or more ACEs in Europe; and 21·0% with one ACE and 31·5% with two or more ACEs in north America) would result in a corresponding reduction of 3 million DALYs (approximately 2 million in Europe and 1 million in north America) equivalent to annual savings of $105 billion ($49 billion in Europe, $56 billion in north America).

**Figure 2:**
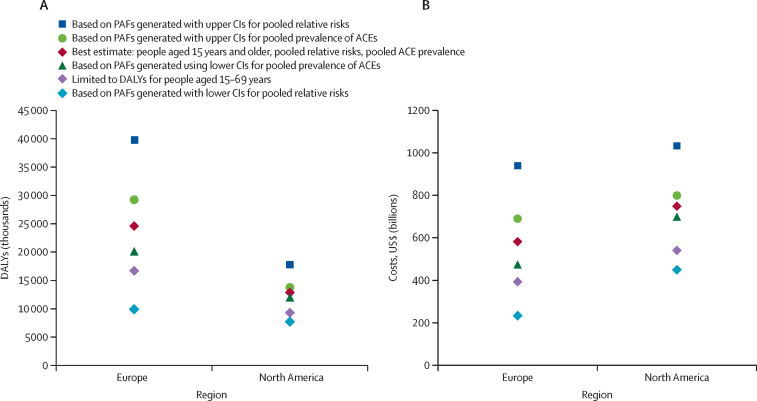
Sensitivity analyses of combined ACE-attributable DALYs and costs relating to risk factors and causes of ill health (A) ACE-attributable DALYs. (B) ACE-attributable costs. DALYs for risk factors (harmful alcohol use, illicit drug use, smoking, and obesity) exclude those attributed to causes of ill health (anxiety, depression, cancer, cardiovascular disease, diabetes, and respiratory disease). PAF=population-attributable fraction.

In sensitivity analyses, we also generated regional PAFs for each outcome by use of upper and lower 95% CIs for pooled RRs and ACE prevalence (figure 2; appendix p 4). After excluding duplicated DALYs, we estimated total annual costs from ACEs for the lowest costed model at $233 billion for Europe (equivalent to 1·07% of regional GDP) and $448 billion for north America (2·13% of GDP); and, for the highest costed model, at $938 billion for Europe (4·31% regional GDP) and more than $1 trillion for north America (4·90% of GDP).

A sensitivity analysis in which we excluded the four samples that were not from the general population showed variable effects on PAFs by risk factor and cause (appendix p 4). A final sensitivity analysis restricted DALYs to those experienced by individuals aged 15–69 years. Because many long-term conditions affect individuals older than 70 years, this adjustment resulted in a substantive effect on estimates, reducing total DALYs attributable to ACEs by 32·1% in Europe and 27·8% in north America, and decreasing estimated costs to $394 billion in Europe and $540 billion in north America.

## Discussion

Several studies^1^ have examined how ACEs relate to the adoption of health-harming behaviours and development of ill health. We combined such studies to estimate the annual health and financial costs accrued because of ACEs. These costs appear substantive, totalling 37·5 million DALYs and $1·3 trillion per annum across north America and Europe for four risk factors and six causes of ill health. We also examined costs associated with experiencing multiple ACEs compared with just one. Across all outcomes, experience of two or more ACEs resulted in higher RRs of risk factors and causes of ill health and accounted for a greater proportion of DALYs and costs than one ACE. Reducing such costs requires work that connects child maltreatment, domestic violence, caregiver mental illness, and other sources of childhood trauma. However, these issues have traditionally been addressed and economically assessed separately.^11^

Our findings support a strong relationship between ACEs and conditions relating to mental health. PAFs associated with ACEs for depression, anxiety, and illicit drug use ranged from 27·5% to 41·1%. The burden of disease related to mental illness is growing globally,^42^ and our findings reinforce previous results that identify childhood as a crucial time in establishing the foundations of good mental health.^43^ Although NCDs had lower PAFs to ACEs than mental health conditions (ranging from 7·5% to 27·6%; table 3), they nevertheless reflect huge, ongoing, and avoidable costs to the health and economies of both regions. ACE-related cancer, cardiovascular disease, respiratory disease, and diabetes together accounted for an estimated annual 15·5 million DALYs in Europe and 7 million DALYs in north America. Studies^25^ linking ACEs to NCDs have been available for more than two decades. However, global efforts to tackle risk factors for NCDs have typically focused on addressing alcohol, tobacco, and food consumption directly rather than childhood stressors that might leave individuals vulnerable to harmful consumption of such items. Our study has identified that potentially 319 million adolescents and adults (15 years and older) across Europe and 172 million in north America could have a legacy of ACEs, with 142 million individuals in Europe and 103 million individuals in north America having experienced multiple ACEs. Each individual could carry ACE-related vulnerabilities to health-harming behaviours and, ultimately, NCDs. Global and national NCD plans need to be cognisant that ACEs contribute considerably to risk.

Even a modest 10% reduction in the prevalence of individuals with single or multiple ACEs in both regions could be equivalent to annual savings of $105 billion (3 million DALYs). Evidence-based approaches to prevent ACEs are available, including programmes to detect and support families at risk.^44^, ^45^ For those experiencing ACEs, interventions to improve parenting skills, to strengthen parent–child attachment, and to develop children's resilience can help to moderate the harmful effects of ACEs.^46^ However, advocacy for investment in such programmes rarely factors in the long-term benefits to mental health and NCD prevention. Consequently, they underestimate their potential return on investment and strategic appeal. Our results suggest that many conditions commonly seen in adults by health professionals are rooted in childhood. Trauma-informed health care^47^ can ensure such individuals receive support to address underlying drivers of health-harming behaviours and resultant NCDs. However, training on ACEs is rarely part of health-care and social-care curricula.

ACEs are relatively common events, even in the predominantly high-income and upper-middle-income countries included in this study. Additional ACE surveys are required to address the paucity of information on populations outside of north America and Europe. However, a growing body of research in low-income and middle-income countries (LMIC) already suggests that ACEs are more widespread in some parts of the world and show similar cumulative effects on health.^9^, ^48^ Further research is required on what constitutes an ACE, particularly in LMICs where, for instance, millions of children are exposed to conflict, community-based violence, and other stressful experiences. Since marginalised communities in both high-income countries and LMICs often experience disproportionate numbers of ACEs, research is also required to ensure ACEs in such communities are fully represented in future estimates of the total health and economic burden of ACEs. A combination of ACE prevention, resilience building, and trauma-informed support could substantially reduce costs associated with ACEs. However, poor investment in interventions that provide safe and nurturing childhoods and help to deliver healthier, better educated, and more prosperous citizens represents short-termism in the thinking of governments and society at large. Such thinking might be costing around 3% of GDP, or considerably more if other ACE-related causes of ill health (such as violence), social issues (such as unemployment), and crime-related costs (such as imprisonment) are also factored in.

Our study has some limitations. The analysis was limited to risk factors and causes of ill health where suitable ACE studies had been done. Although these factors included many major risks to health and pressures on health-care systems, our estimates of ACE-related DALYs and costs are incomplete. Further, inconsistency remains in the definitions and types of ACEs and health outcomes measured, study design, and methods of analysis. All such factors could affect the pooled estimates and associated heterogeneity. Heterogeneity varied considerably between regions, conditions, and extent of ACE exposure (table 2), and more consistent studies are required to improve confidence in the types of population-level estimates that we calculated. However, although PAFs differed between north America and Europe, we found consistencies in the absolute value of some PAFs to ACEs (eg, for smoking) and the relative size of others (eg, higher PAFs for mental health conditions). ACE prevalence also varied between regions: prevalence was higher in north American populations, even when similar approaches were used with population samples. Previous work^9^ has identified a higher prevalence of violence against children in north America than in Europe. Although beyond the scope of this study, this finding might reflect societal differences in health, social care, and criminal justice environments.

The studies we included used binary measures of ACE exposure, which do not account for age and length of exposure. Such factors are likely to be important in refining relationships between ACEs and health over the life course. Categorisation of ACEs varied and so we were restricted to analysing in categories of zero, one, or two or more ACEs preventing more detailed examination of ACE counts and combinations. Standardisation of categorisation and other ACE analytical procedures would help to better elucidate dose-response relationships between ACEs, disease risk, and DALYs in future meta-analyses. Many studies were retrospective and self-reported, excluding individuals who had died (potentially from ACE-related conditions) and leaving participants subject to recall bias. Thus, additional prospective (as well as retrospective) ACE studies are an important consideration for further research. Depression might affect recall of ACEs although, generally, false-positive reports of ACEs appear rare.^49^ Study inclusion was restricted to general populations and those not at high risk, but individuals unable to take part in general surveys (such as homeless people) are likely to have a high number of ACEs.^50^ Health outcomes were largely self-reported and study definitions were often absent or could not be matched to GBD categories. Further, overlap inevitably exists between DALYs for risk factors and causes. We accounted for this overlap by excluding risk factor DALYs captured in causes before calculating DALY totals. Although most studies controlled for age and socioeconomic groups, four studies^22^, ^28^, ^35^, ^36^ did not account for both (table 1). We did not adjust our analysis for study quality, but one study^35^ consistently reported lower risk estimates. Where studies controlled for socioeconomic groups, we used adjusted risks. However, three studies^20^, ^28^, ^31^ included a socioeconomic factor (eg, household living on public assistance) as an ACE. Poor consistency in how ACE-related risk measures are adjusted for socioeconomic groups is a limitation of ACE research and a likely source of heterogeneity. More standardisation of socioeconomic and ACE measurements would help to identify both the relationships each have with outcomes of interest and the relationships between ACEs and deprivation. Such understanding is crucial if policy is to appropriately address the relative contributions that poverty and ACEs make to poor life course health.

Sensitivity analyses generated a range of costs, but even the lower estimates represent substantive areas for potential savings through tackling ACEs and their consequences. Estimated costs are based on a broad assumption that a DALY might be considered equivalent to a year's GDP per capita.^15^, ^41^ This human capital model is likely to be conservative (by not fully accounting for pain and suffering) and more detailed economic modelling is required as better ACE-related data emerges. Finally, although measured where possible (appendix pp 8, 12, 16, 21) the potential effects of publication bias cannot be discounted.

ACEs were first described in the 1990s as a combined measure of childhood adversity.^25^ During the past two decades, this combined measure and related terminology have continued to develop, which might have affected what has been captured and included in this meta-analysis. During the same period, ACE studies have increasingly provided a framework for examining the health effects of childhood trauma across the life course. Locally, findings from individual studies have been used to advocate for multi-agency actions and policies to prevent ACEs and support those suffering their consequences.^51^, ^52^ Sadly, however, the effects of ACEs on national and international policy has been restricted. Our data from studies across Europe and north America show the huge personal and economic burden associated with children experiencing several ACEs. These burdens can be avoided if governments prioritise investment in children and fund evidence-based prevention strategies.^53^ The eradication of child maltreatment is a reasonable goal for the 21st century. However, it requires an escalation of action to coordinate activities by health, social, judicial, educational, and other departments to collectively address child maltreatment, substance use, domestic violence, and other factors that deprive children of safe and nurturing childhoods. Arguably, such actions are justified simply from the perspective of protecting child rights. However, our results suggest that tackling ACEs will also reduce pressures from NCDs and mental illnesses on health systems, societal harms from alcohol and drug use, and contribute substantively to the economic development of nations.
