# Supplementary material for: Life course health consequences and associated annual costs of adverse childhood experiences across Europe and North America: a systematic review and meta-analysis
Source: Lancet Public Health. 2019 Sep 3;4(10):e517–28. doi: 10.1016/S2468-2667(19)30145-8 (PMC7098477; doi:10.1016/S2468-2667(19)30145-8)
Supplement: Supplementary appendix [file mmc1.pdf]

THE LANCET
Public Health

Supplementary appendix

This appendix formed part of the original submission and has been peer reviewed. We post it as supplied by the authors.

Supplement to: Bellis MA, Hughes K, Ford K, Rodriguez GR, Sethi D, Passmore J. Life course health consequences and associated annual costs of adverse childhood experiences across Europe and North America: a systematic review and meta-analysis. *Lancet Public Health* 2019; published online Sept 13. http://dx.doi.org/10.1016/S2468-2667(19)30145-8.

Appendix

Table A1: Countries in each region

Europe*	North America
Albania, Armenia, Andorra, Austria, Azerbaijan, Belarus, Belgium, Bosnia and Herzegovina, Bulgaria, Croatia, Cyprus, Czech Republic, Denmark, Estonia, Finland, France, Georgia, Germany, Greece, Hungary, Iceland, Ireland, Israel, Italy, Kazakhstan, Kyrgyz Republic, Latvia, Lithuania, Luxembourg, The former Yugoslav Republic of Macedonia, Malta, Moldova, Montenegro, Netherlands, Norway, Poland, Portugal, Romania, Russian Federation, Serbia, Slovak Republic, Slovenia, Spain, Switzerland, Sweden, Tajikistan, Turkey, Turkmenistan, Ukraine, United Kingdom, Uzbekistan	Bermuda, Canada, United States

*Monaco and San Marino excluded due to lack of GBD data availability.

Table A2: Study definitions within each outcome category

Outcome	GBD matched (ID)	Study definitions (number of samples, time frame)	References
**Risk factors**			
Harmful alcohol use	Alcohol use (102)	Self–reported alcoholic (1, L); Self–reported problem drinker or alcoholic (1, L); Six or more standard drinks on one occasion at least weekly (2, C); Binge drinking (2, NR); >2 (male)/>1 (female) drinks daily (1, C); AUDIT C score 5+ (1, C); AUDIT C score ≥9 (1, NR)	[1–9]
Illicit drug use	Drug use (103)	Street drug use (2, L); Cannabis use (3, L)	[1–4,7]
Smoking	Smoking (99)	Smoker (8, C); Daily smoking (3, C)	[1–11]
Obesity	High body mass index (108)	BMI >25 (1, C); BMI ≥25 (1, C); BMI ≥30 (3, C); BMI ≥35 (1, C); BMI ≥40 (1, C)	[1,3,5–7,11,12]
**Causes**			
Anxiety	Anxiety disorders (571)	Anxiety (1, L); GAD–2 (1, C); Trouble with nervousness (1, NR); Self–reported treatment for anxiety (1, L)	[1,8,9,13]
Depression	Major depressive disorder (568)	Medically recorded treatment for: depression (1, over 7 year period in adulthood), Unipolar depressive disorder (1, age 15–34 years); Self–reported depressed affect (1, L); Self–reported diagnosis for depression (2; L); Self–reported depression (1, C); Depressive symptoms: (1, C, PHQ–9; 1, C, PHQ–2); Depression (1, L)	[1,5,6,8,9,11,13–16]
Cancer	Neoplasms (410)	Any cancer (6, L)	[6,7,17–20]
Cardiovascular disease	Cardiovascular diseases (491)	Cardiovascular disease (4, L); Heart disease (including coronary heart disease and ischaemic heart disease; 4, L); Heart attack (1, L)	[5–7,9,11,12,17,20,21]
Diabetes	Diabetes mellitus (587)	Diabetes (5, L; 1, NR); Type 2 diabetes (2, L); Diabetes or high blood sugar (1; treatment in last 12 months)	[5–7,9,11,12,17,18,20]
Respiratory disease	Chronic respiratory diseases (508)	Respiratory disease (3, L); COPD (2, L; 1, NR); Chronic bronchitis, emphysema, or COPD (1, L); Chronic bronchitis or emphysema (1, L); Lung disease (1, L); Asthma (2, L)	[3,6,7,9,11,17,18,20,22,23]

AUDIT=Alcohol Use Disorders Identification Test; BMI=Body Mass Index; C=current; COPD=Chronic obstructive pulmonary disease; GAD=General Anxiety Disorder; GBD=Global Burden of Disease; ID=Identification; L=lifetime; NR=not reported; PHQ=Patient Health Questionnaire.

Panel A*. Adverse childhood experience categories measured by included studies

Type of childhood adversity	Number of studies
Physical abuse	20
Household substance abuse	22
Sexual abuse	19
Household mental illness	19
Exposure to domestic violence	16
Emotional/psychological/verbal abuse	17
Parental separation/divorce	19
Household member incarcerated/criminality	19
Neglect	8
Parental death	3
Child welfare intervention (e.g. out of home care)	3

Other types (measured in <3 studies) include:
Family welfare/financial difficulties; residential instability; parental illness; parental disability; separation from mother; frightening experience; sent away from home; child incarcerated; educational difficulties (e.g. school drop–out); lost home to fire, flood or natural disaster

*Questions used to determine ACEs varied between studies.

Table A3: DALYs for risk factors, excluding DALYs for causes of ill health studied*

Risk factor	Region	DALYs (thousands) excluding those for causes studied*	ACE–attributable DALYs (thousands) excluding those for causes studied*			ACE–attributable costs (US$ billions) excluding those for causes studied*		
			1 ACE	≥2ACEs	All ACEs	1 ACE	≥2ACEs	All ACEs
Harmful alcohol use	Europe	14880	1347	2325	3673	32	55	87
	North America	3708	278	758	1036	16	44	60
Illicit drug use	Europe	5333	571	1247	1818	13	29	43
	North America	6833	497	2308	2806	29	134	163
Smoking	Europe	6682	371	845	1216	9	20	29
	North America	1657	69	325	393	4	19	23
Obesity	Europe	4647	66	193	260	2	5	6
	North America	2740	45	200	246	3	12	14

ACE=Adverse childhood experience; DALY=Disability Adjusted Life Year; *Anxiety, depression, cancer, cardiovascular disease, respiratory disease, diabetes.

Table A4: Sensitivity analysis: ACE–attributable DALYs (thousands) for risk factors, causes and summed totals

		Best estimate (Age 15+, pooled relative risks, pooled ACE prevalence)			Based on PAFs generated using lower CIs for pooled relative risks			Based on PAFs generated using upper CIs for pooled relative risks			Based on PAFs generated using lower CIs for pooled prevalence of ACEs			Based on PAFs generated using upper CIs for pooled prevalence of ACEs			Limited to DALYs for 15–69 year olds			Based on PAFs generated using data from general population samples only†		
		ACEs			ACEs			ACEs			ACEs			ACEs			ACEs			ACEs		
**Risk factors**	**Region**	**1**	**≥2**	**All**	**1**	**≥2**	**All**	**1**	**≥2**	**All**	**1**	**≥2**	**All**	**1**	**≥2**	**All**	**1**	**≥2**	**All**	**1**	**≥2**	**All**
Harmful alcohol use	Eur	2217	3825	6042	1188	534	1722	2848	7694	10542	1869	3157	5026	2551	4480	7031	1938	3344	5282	1838	2181	4019
	NA	335	915	1250	183	310	493	442	1494	1935	323	848	1171	347	980	1326	317	865	1182	270	644	914
Illicit drug use	Eur	610	1333	1944	451	1258	1709	772	1402	2174	526	1124	1650	688	1530	2218	574	1255	1829	611	1282	1892
	NA ‡	514	2385	2899	379	2191	2571	644	2567	3211	502	2239	2741	525	2523	3048	496	2301	2797	–	–	–
Smoking	Eur	2142	4873	7015	1601	4316	5917	2680	5429	8110	1781	3966	5747	2499	5788	8288	1513	3443	4956	1480	5078	6558
	NA	479	2274	2753	88	1625	1712	850	2883	3732	461	2102	2563	498	2442	2940	296	1405	1701	520	2277	2797
Obesity	Eur	430	1251	1681	–85	–858	–942	874	3763	4638	348	991	1338	516	1529	2044	279	812	1091	413	1187	1599
	NA	207	917	1124	–11	256	244	409	1577	1986	196	837	1033	218	998	1216	141	623	764	118	644	762
**Risk factors, excluding DALYs for causes of ill health studied***																						
Harmful alcohol use	Eur	1347	2325	3673	722	325	1047	1731	4677	6408	1136	1919	3055	1550	2724	4274	1233	2127	3360	1117	1326	2443
	NA	278	758	1036	151	257	409	366	1238	1604	268	703	970	287	812	1099	256	698	953	224	534	758
Illicit drug use	Eur	571	1247	1818	422	1177	1598	722	1311	2034	492	1052	1543	643	1431	2074	547	1195	1741	571	1199	1770
	NA	497	2308	2806	367	2121	2488	623	2485	3108	486	2168	2653	508	2442	2950	484	2245	2728	–	–	–
Smoking	Eur	371	845	1216	277	748	1026	465	941	1406	309	688	996	433	1004	1437	271	616	886	257	880	1137
	NA	69	325	393	13	232	245	121	412	533	66	300	366	71	349	420	44	210	255	74	325	400
Obesity	Eur	66	193	260	–13	–133	–146	135	582	717	54	153	207	80	236	316	34	98	131	64	183	247
	NA	45	200	246	–2	56	53	89	345	434	43	183	226	48	218	266	25	112	138	26	141	167
**Causes**																						
Cancer	Eur	716	4258	4974	–1057	2523	1466	2499	5999	8498	586	3415	4001	847	5134	5981	475	2826	3300	689	4052	4741
	NA	351	1290	1641	–181	552	370	872	1998	2870	333	1178	1511	369	1403	1772	225	827	1052	295	1189	1483
Cardiovascular disease	Eur	1492	6357	7848	–218	2454	2236	3059	10591	13650	1223	5102	6324	1765	7660	9425	738	3144	3882	1437	6049	7486
	NA	496	2825	3321	131	2091	2222	841	3536	4378	475	2603	3077	516	3045	3561	258	1469	1727	546	2894	3439
Respiratory disease	Eur	383	1591	1974	99	763	862	624	2492	3116	319	1296	1615	446	1888	2334	211	878	1089	609	1839	2448
	NA	298	1410	1707	197	1007	1204	384	1802	2185	287	1307	1595	308	1509	1817	159	752	911	268	1280	1549

Diabetes	Eur	41	636	677	–244	185	–59	314	1117	1432	33	506	539	49	773	821	26	409	436	39	604	643
	NA	104	209	313	16	–39	–23	185	457	643	99	190	289	110	228	338	71	142	213	115	199	314
Depression	Eur	406	807	1213	393	736	1129	419	878	1297	345	670	1015	464	939	1403	344	684	1029	394	774	1168
	NA	95	703	798	49	562	611	134	833	967	93	660	753	97	743	840	89	662	751	78	695	774
Anxiety	Eur ǂ	252	714	966	106	604	711	396	817	1213	215	594	809	288	829	1117	224	633	857	245	685	930
	NA	25	597	622	–150	276	127	172	857	1029	24	557	581	25	636	662	23	552	575	8	664	672
Summed total (DALYs for causes plus DALYs for risk factors excluding those for causes of ill health studied*)																						
	Eur	5645	18973	24618	488	9382	9870	10365	29406	39771	4710	15395	20105	6567	22616	29183	4102	12610	16712	5423	17590	23013
	NA	2257	10626	12883	591	7115	7706	3789	13963	17751	2173	9848	12021	2340	11386	13726	1634	7669	9303	–	–	–

ACE=Adverse childhood experience; CI=Confidence interval; DALY=Disability Adjusted Life Year; Eur=European studies; NA=North American studies; na=not applicable, no general population studies available for illicit drug use and consequently summed total cannot be calculated; PAFs=Population attributable fractions. *Anxiety, depression, cancer, cardiovascular disease, respiratory disease, diabetes. †Excluding Anda et al, 2006[1], Bellis et al 2014[4], Dong et al, 2004[21], Felitti et al, 1998[18], Poole et al, 2017[16], Wainwright et al, 2001[23]. ǂBased on a single study.

Webfigure 1a: Prevalence of 1 ACE in European studies: summary meta–analysis plot (random effects)

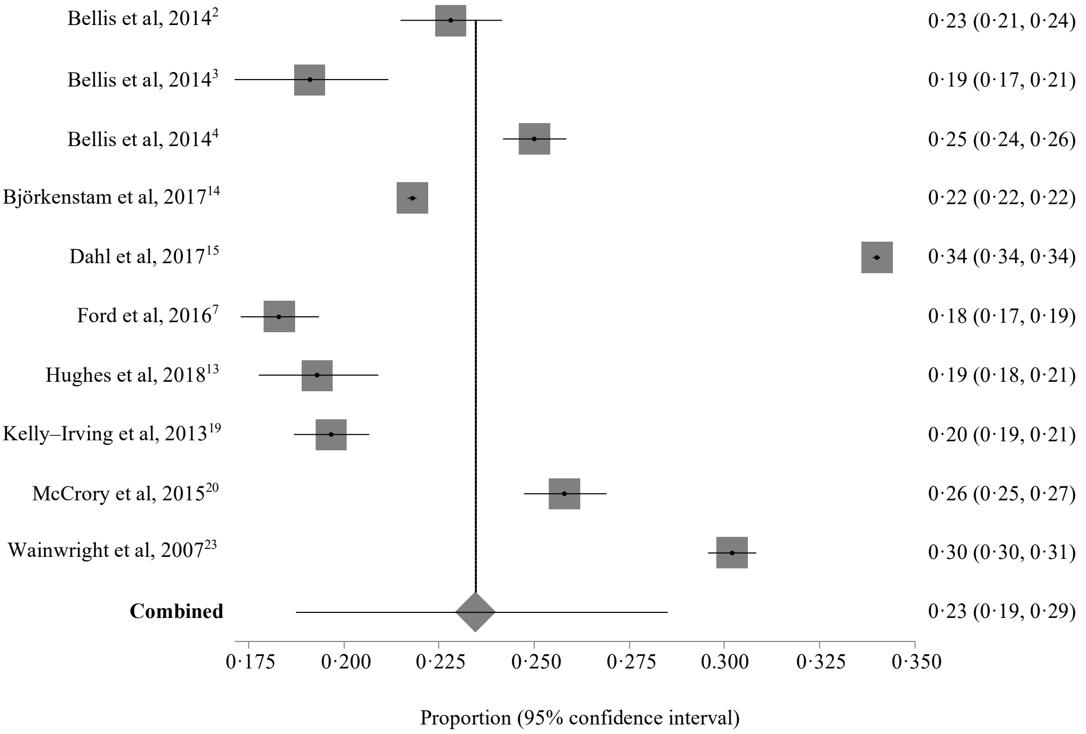

All studies: I^2 = 100% (95% CI = 100% to 100%); Cochran Q = 25,265·503483 (df = 9) P < 0·0001.

Webfigure 1b: Prevalence of ≥ 2ACEs in European studies: summary meta–analysis plot (random effects)

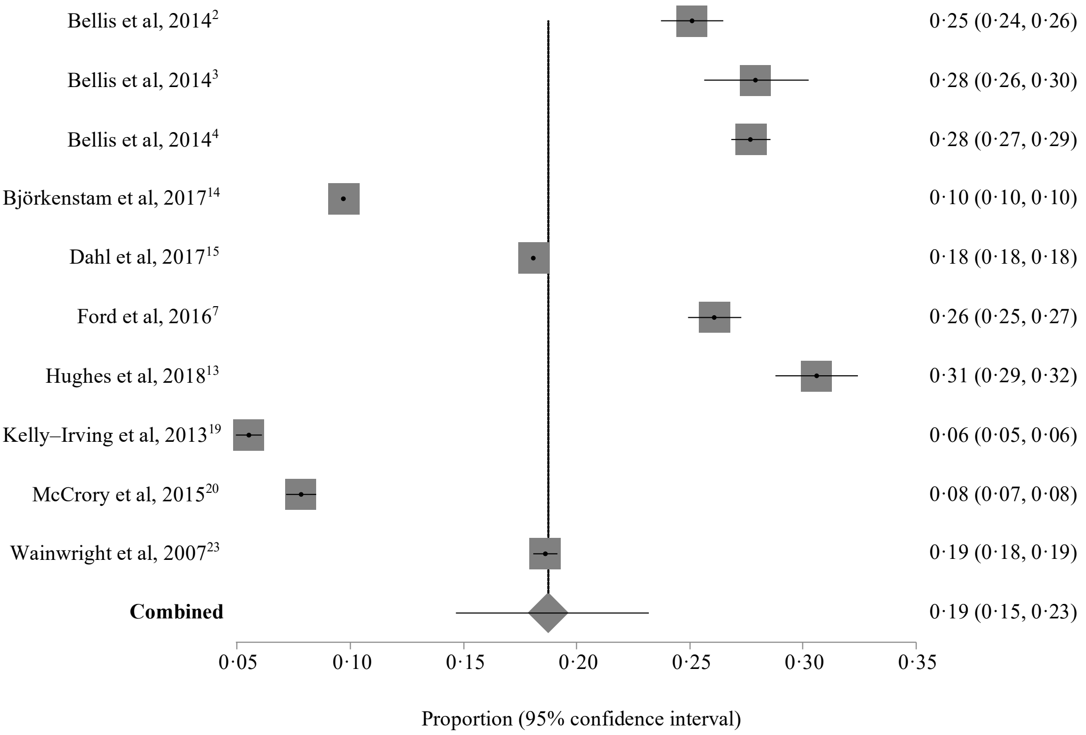

All studies: I^2 = 100% (95% CI = 100% to 100%); Cochran Q = 22,688·577044 (df = 9) P < 0·0001.

Webfigure 2a: Prevalence of 1 ACE in North American studies: summary meta–analysis plot (random effects)

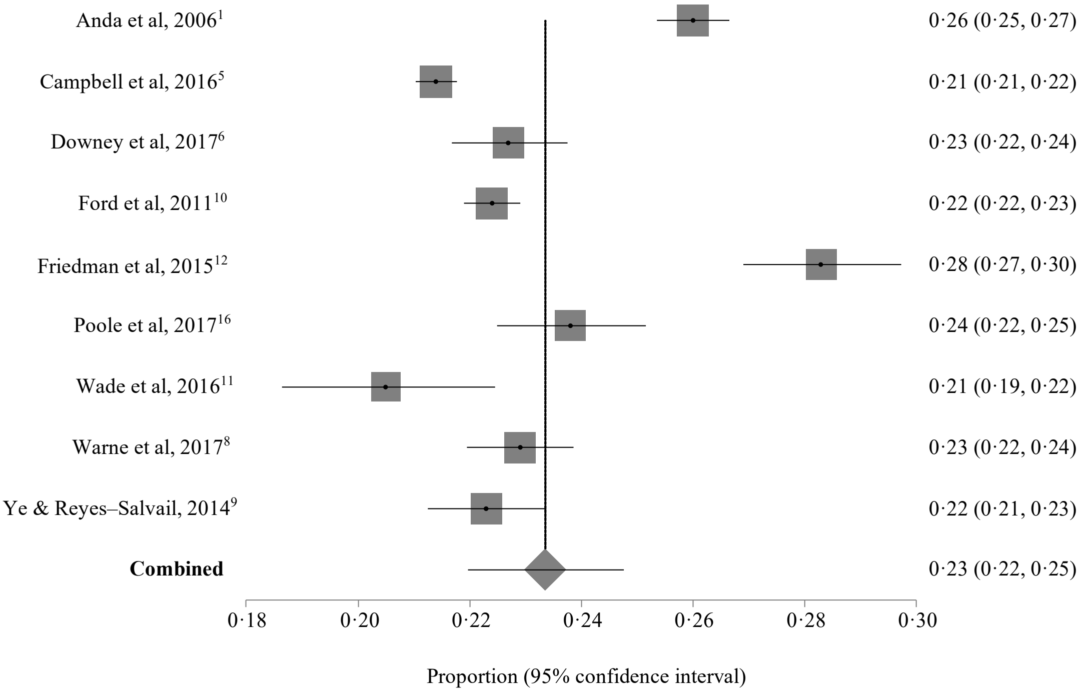

All studies: I^2 = 96·5% (95% CI = 95·3% to 97·2%); Cochran Q = 226·2931 (df = 8) P < 0·0001.

Webfigure 2b: Prevalence of ≥2 ACEs in North American studies: summary meta–analysis plot (random effects)

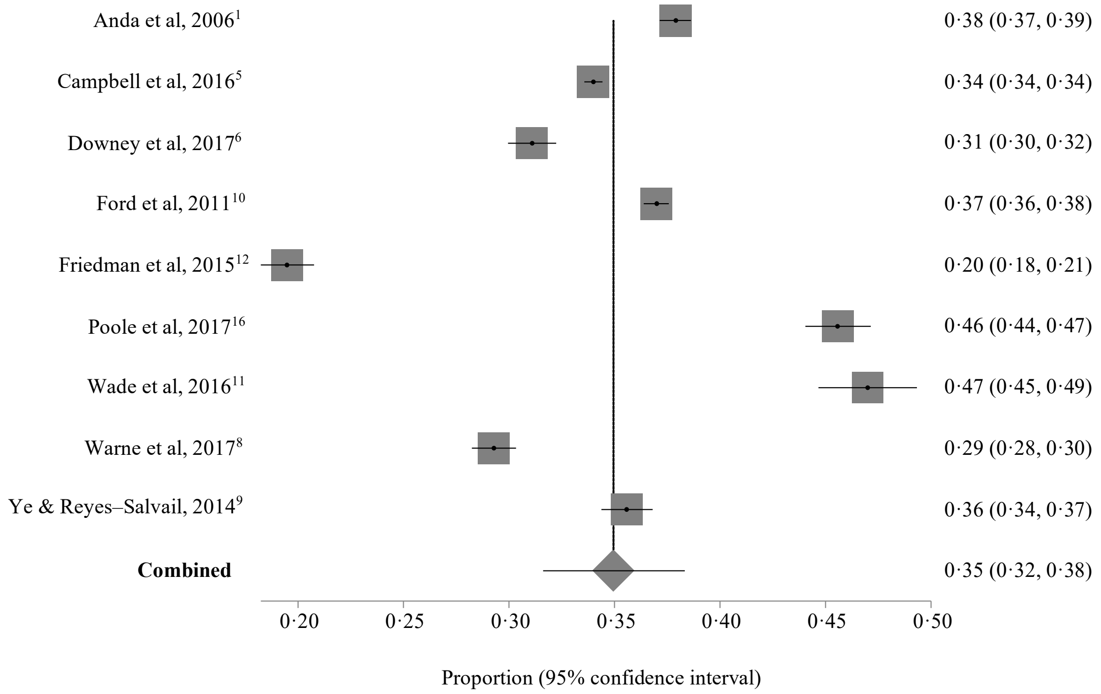

All studies: I^2 = 99·3% (95% CI = 99·2% to 99·3%); Cochran Q = 1,073·641277 (df = 8) P < 0·0001.

Webfigure 3: Prevalence of ACEs: Funnel plot analysis

a) Europe: 1 ACE

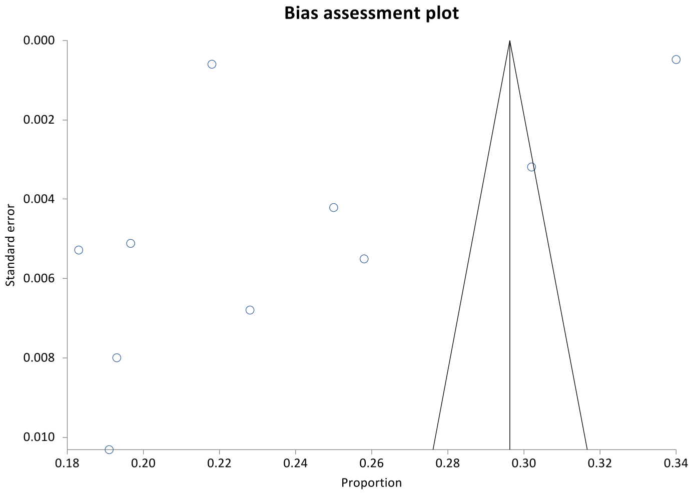

Begg–Mazumdar: Kendall's tau –0·155556, P=0·4843;
Egger: bias –15·697844 (95%CI –67·064191 to 35·668503) P=0·501.

b) Europe: ≥2 ACEs

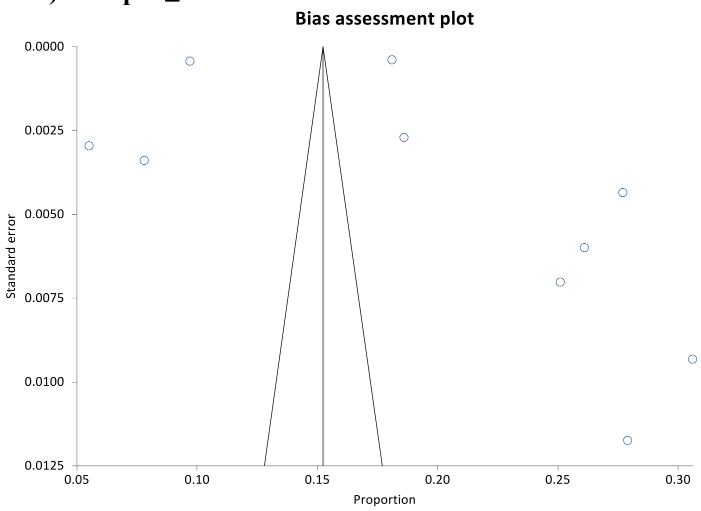

Begg–Mazumdar: Kendall's tau 0·022222, P>0·9999;
Egger: bias 7·589836 (95%CI –42·235722 to 57·415395) P=0·7345.

c) North America: 1 ACE

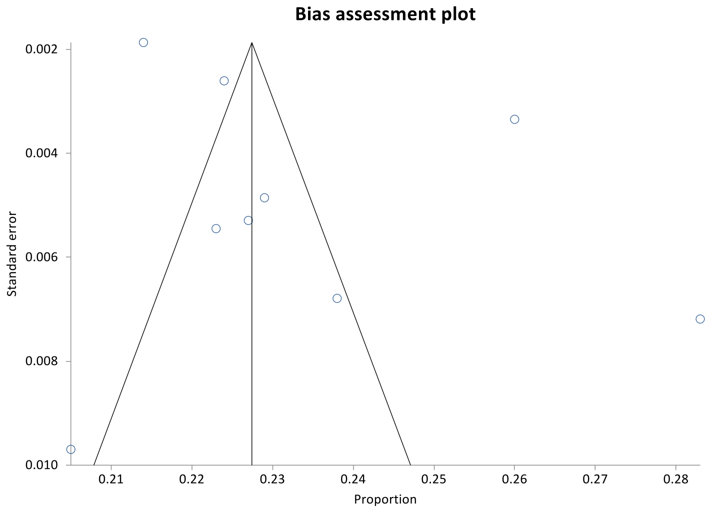

Begg–Mazumdar: Kendall's tau 0·166667, P = 0·6122;
Egger: bias 4·483225 (95% CI = –3·793316 to 12·759766) P = 0·241.

d) North America: ≥2 ACEs

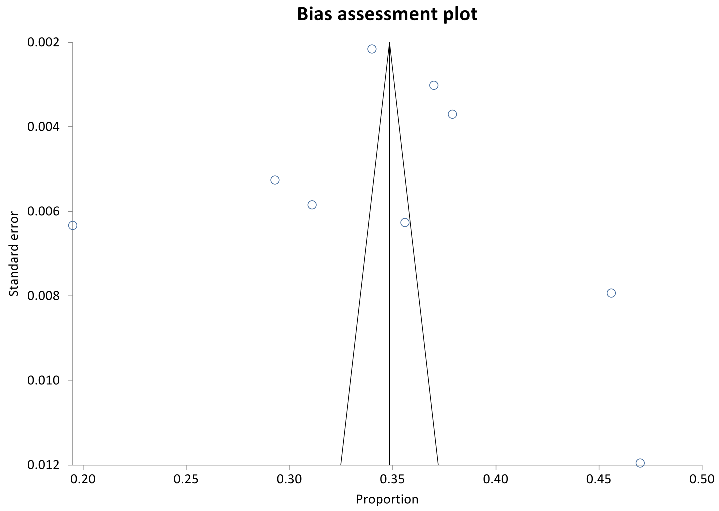

Begg–Mazumdar: Kendall's tau = 0·166667, P = 0·6122;
Egger: bias 0·159589 (95% CI –22·00291 to 22·322087) P=0·9869.

Webfigure 4a: Increased risk of harmful alcohol use with 1 ACE: summary meta–analysis plot (random effects)

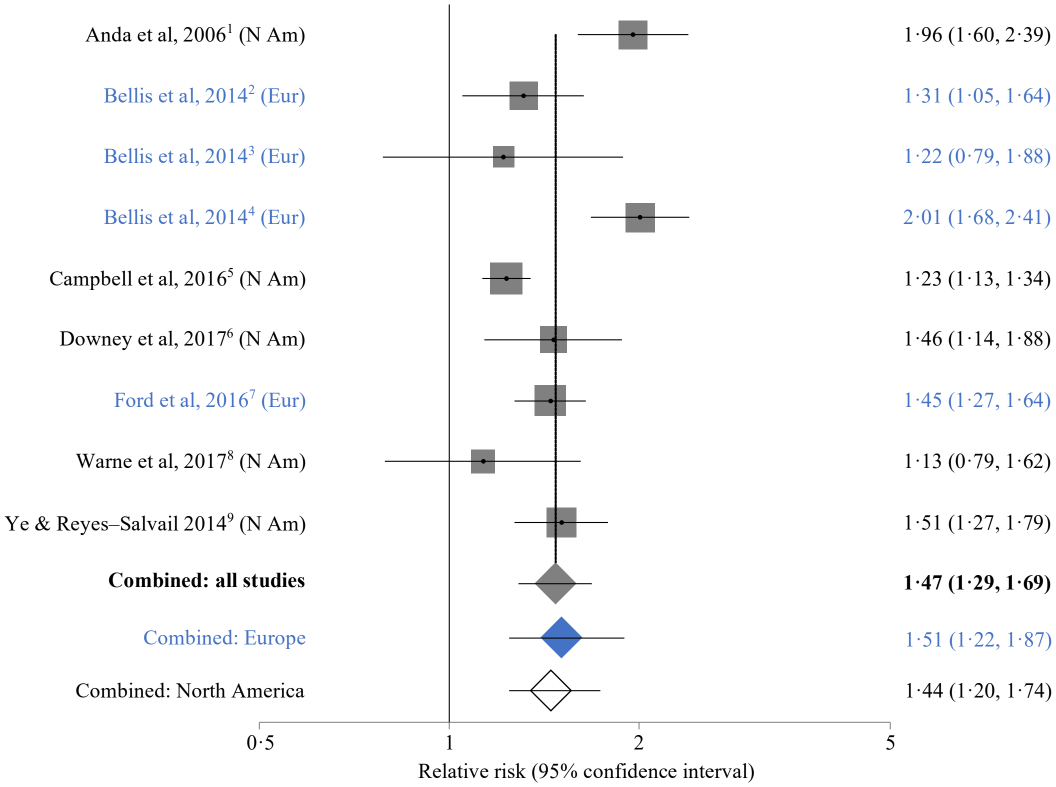

All studies: I^2 = 78·4% (95% CI = 53·9% to 87·1%); Cochran Q = 36·979364 (df = 8) P < 0·0001.
Europe (Eur): I^2 = 75·4% (95% CI = 0% to 89·1%); Cochran Q = 12·215004 (df = 3) P = 0·0067.
North America (N Am): I^2 = 80·1% (95% CI = 37·6% to 89·8%); Cochran Q = 20·13317 (df = 4) P = 0·0005.

Webfigure 4b: Increased risk of harmful alcohol use with ≥2 ACEs: summary meta–analysis plot (random effects)

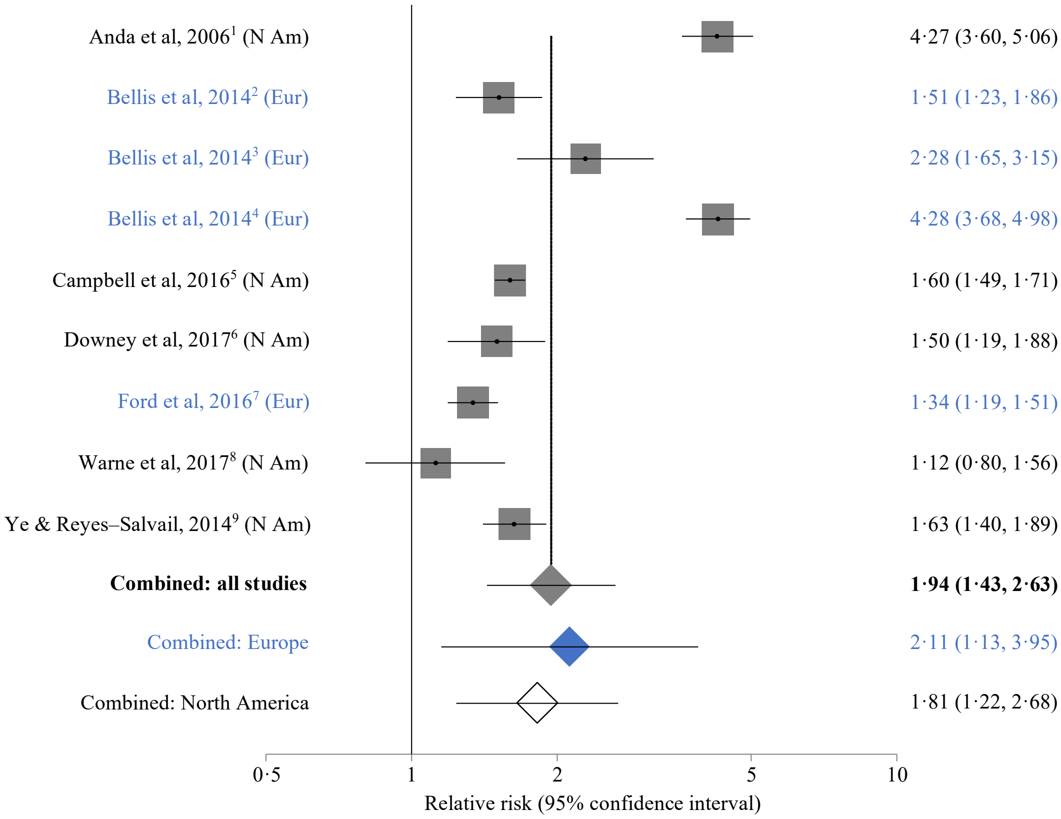

All studies: I^2 = 97·1% (95% CI = 96·3% to 97·7%); Cochran Q = 277·176231 (df = 8) P < 0·0001.
Europe (Eur): I^2 = 98% (95% CI = 97·1% to 98·5%); Cochran Q = 149·404062 (df = 3) P < 0·0001.
North America (N Am): I^2 = 96·7% (95% CI = 95·2% to 97·6%); Cochran Q = 122·940595 (df = 4) P < 0·0001.

Webfigure 5a: Increased risk of illicit drug use with 1 ACE: summary meta–analysis plot (random effects)

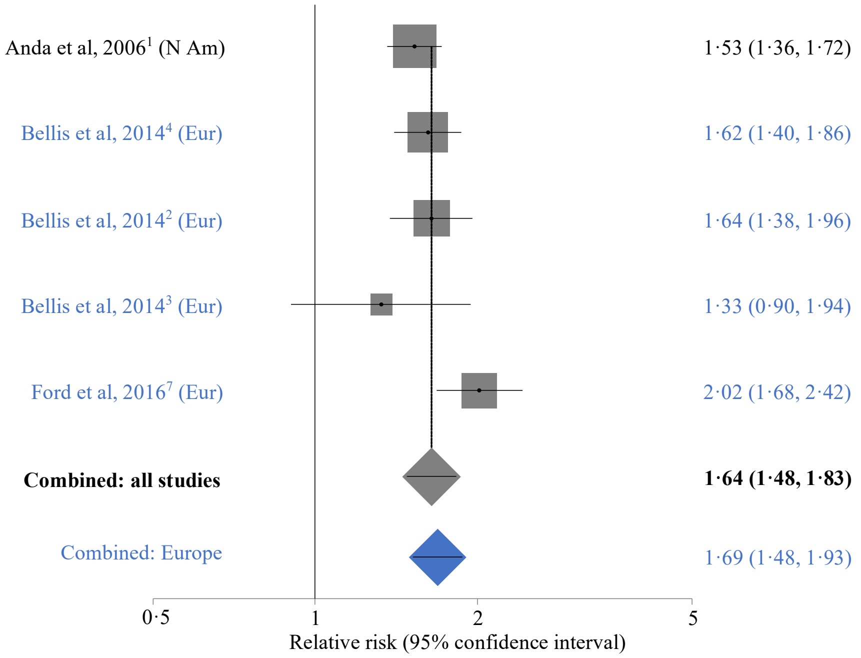

All studies: I^2 = 46·4% (95% CI = 0% to 78·8%); Cochran Q = 7·459456 (df = 4) P = 0·1135.
Europe (Eur): I^2 = 45·7% (95% CI = 0% to 80·7%); Cochran Q = 5·520515 (df = 3) P = 0·1374.

Webfigure 5b: Increased risk of illicit drug use with ≥2 ACEs: summary meta–analysis plot (random effects)

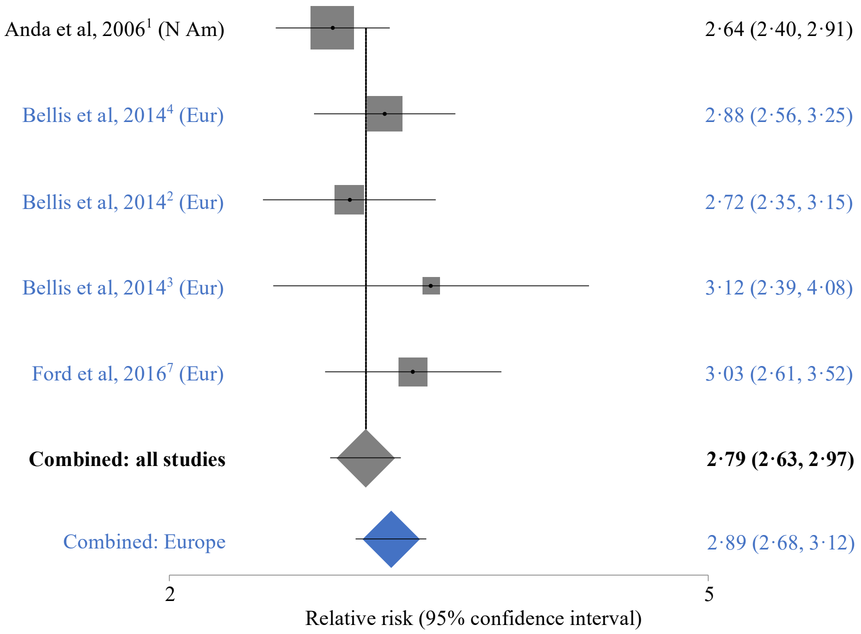

All studies: I^2 = 0% (95% CI = 0% to 64·1%); Cochran Q = 3·501311 (df = 4) P = 0·4777.
Europe (Eur): I^2 = 0% (95% CI = 0% to 67·9%); Cochran Q = 1·357401 (df = 3) P = 0·7155.

Webfigure 6a: Increased risk of smoking with 1 ACE: summary meta–analysis plot (random effects)

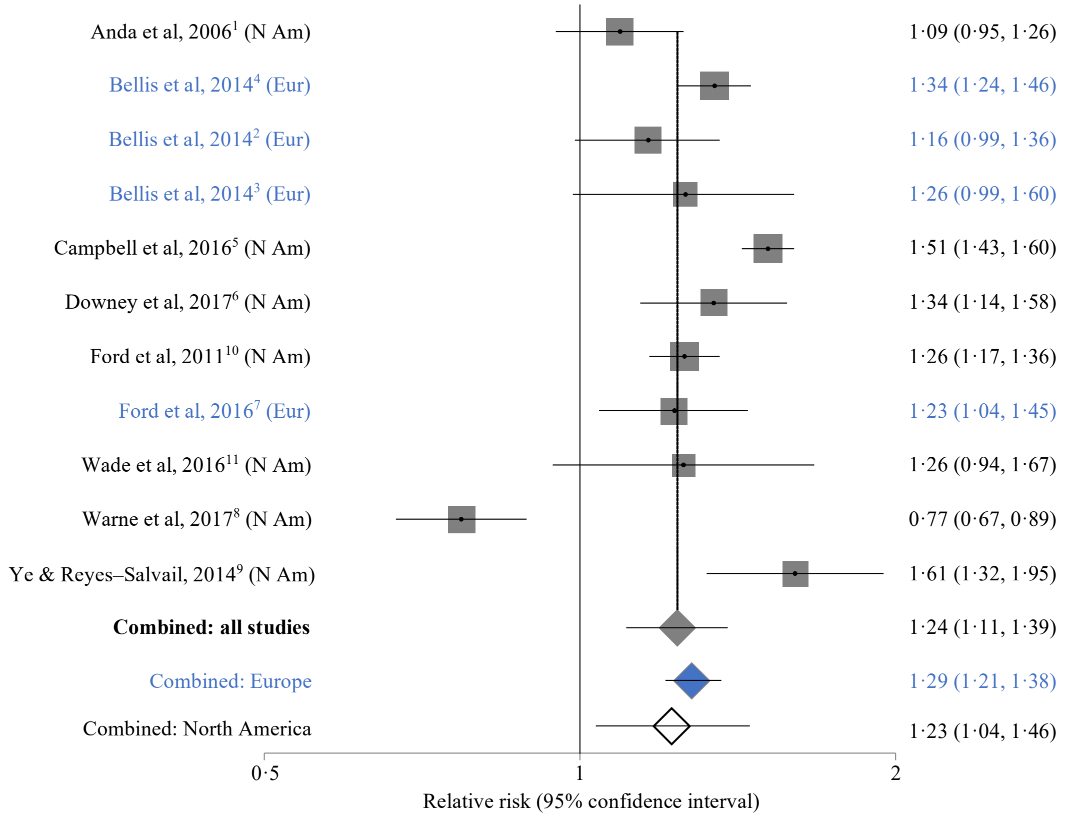

All studies: I^2 = 88·9% (95% CI = 82·5% to 92·3%); Cochran Q = 90·344835 (df = 10) P < 0·0001.

Europe (Eur): I^2 = 1% (95% CI = 0% to 68·2%); Cochran Q = 3·030147 (df = 3) P = 0·387.

North America (N Am): I^2 = 93·1% (95% CI = 88·9% to 95·2%); Cochran Q = 86·921418 (df = 6) P < 0·0001.

Webfigure 6b: Increased risk of smoking with ≥2 ACEs: summary meta–analysis plot (random effects)

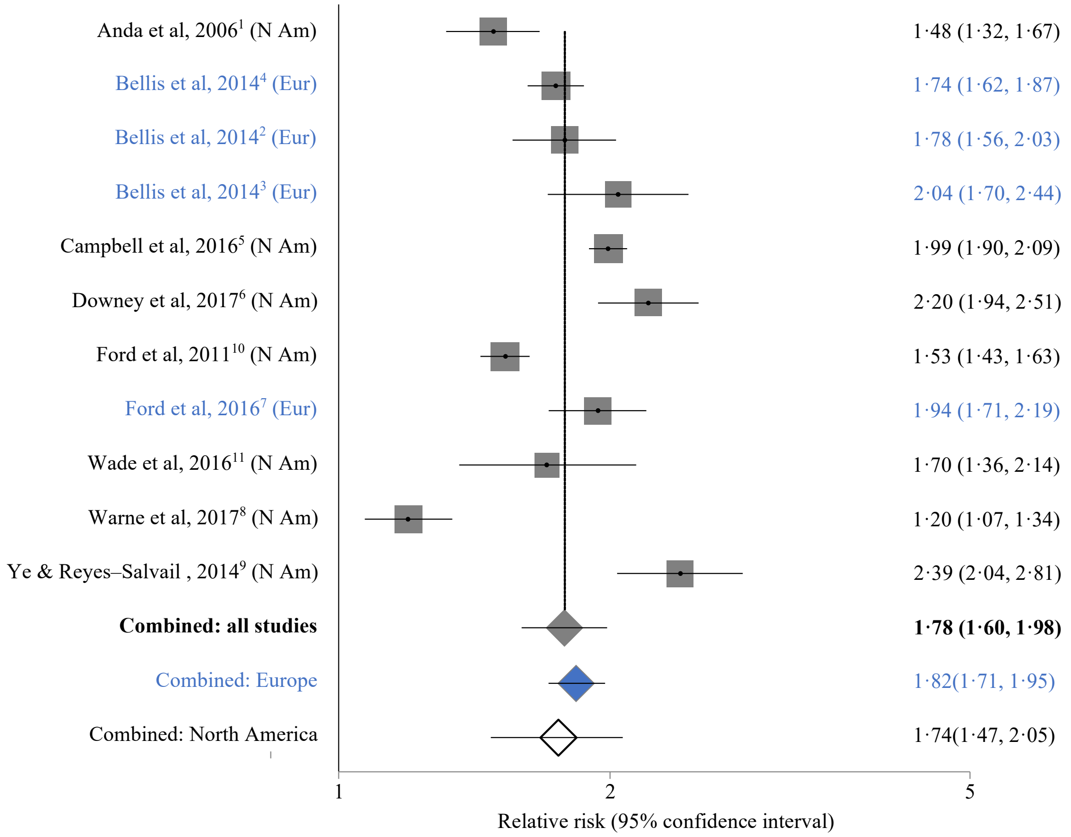

All studies: I^2 = 92·2% (95% CI = 88·5% to 94·2%); Cochran Q = 127·650374 (df = 10) P < 0·0001.

Europe (Eur): I^2 = 25·9% (95% CI = 0% to 75·5%); Cochran Q = 4·051301 (df = 3) P = 0·256.

North America (N Am): I^2 = 95·1% (95% CI = 92·8% to 96·4%); Cochran Q = 122·928275 (df = 6) P < 0·0001.

Webfigure 6c: Increased risk of smoking with 1 ACE: Funnel plot analysis

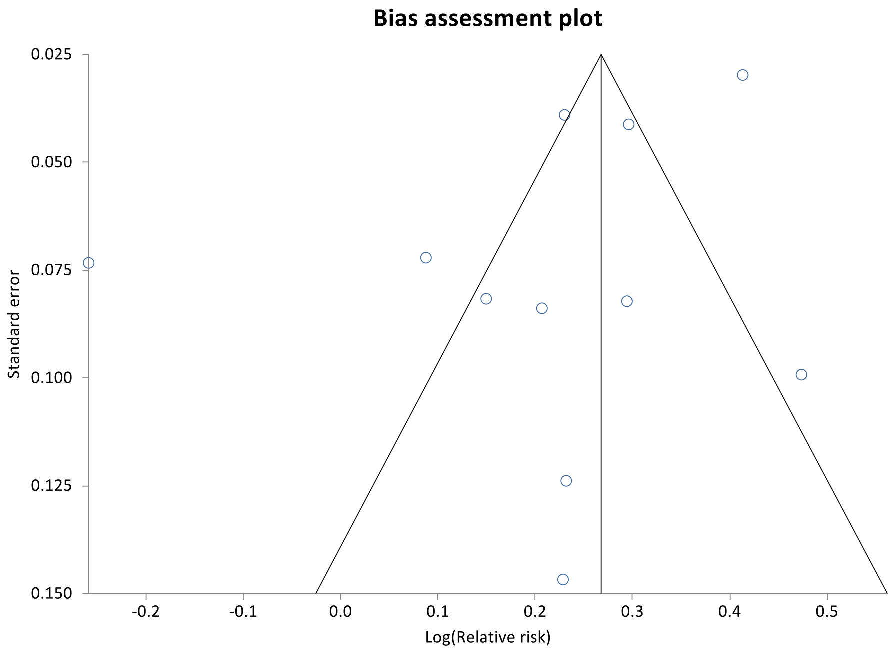

Begg–Mazumdar: Kendall's tau 0·054545, P = 0·8793; Egger: bias –2·762773 (95% CI = –7·020631 to 1·495086) P = 0·1762.

Webfigure 6d: Increased risk of smoking with ≥2 ACEs: Funnel plot analysis

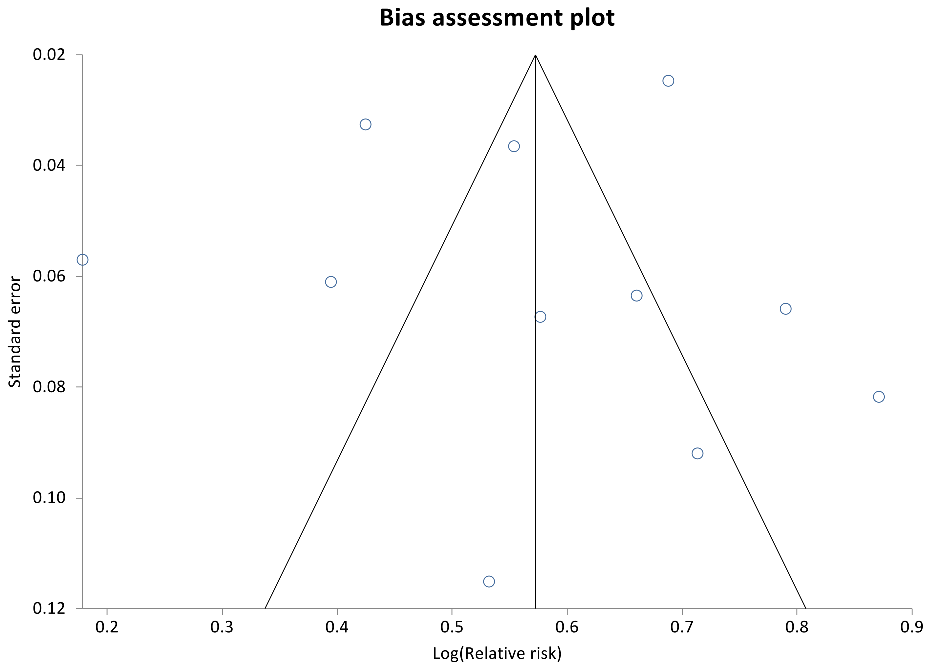

Begg–Mazumdar: Kendall's tau 0·2, P = 0·4454; Egger: bias –0·259826 (95% CI = –6·155866 to 5·636213) P = 0·9228.

Webfigure 7a: Increased risk of obesity with 1 ACE: summary meta–analysis plot (random effects)

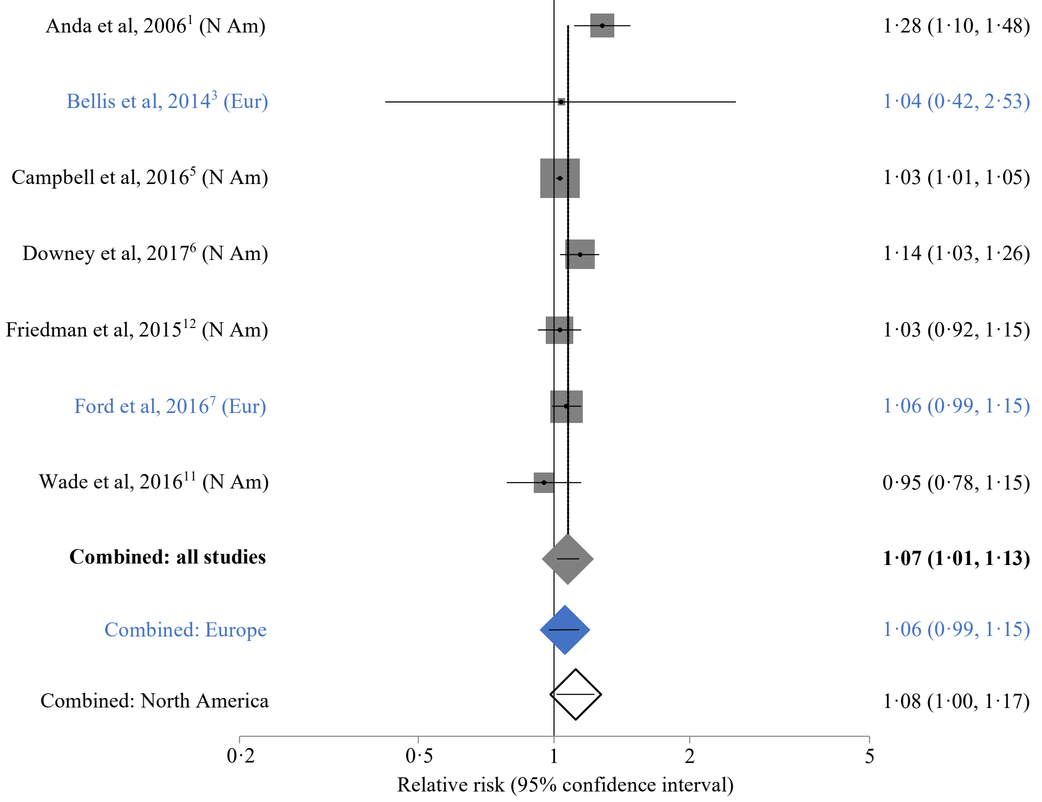

All studies: I^2 = 56·6% (95% CI = 0% to 79·4%); Cochran Q = 13·837732 (df = 6) P = 0·0315.

Europe (Eur): I^2 = 0% (95% CI = *% to *%); Cochran Q = 0·003191 (df = 1) P = 0·955.

North America (N Am): I^2 = 69·9% (95% CI = 0% to 86·2%); Cochran Q = 13·28994 (df = 4) P = 0·0099.

Webfigure 7b: Increased risk of obesity with ≥2 ACEs: summary meta–analysis plot (random effects)

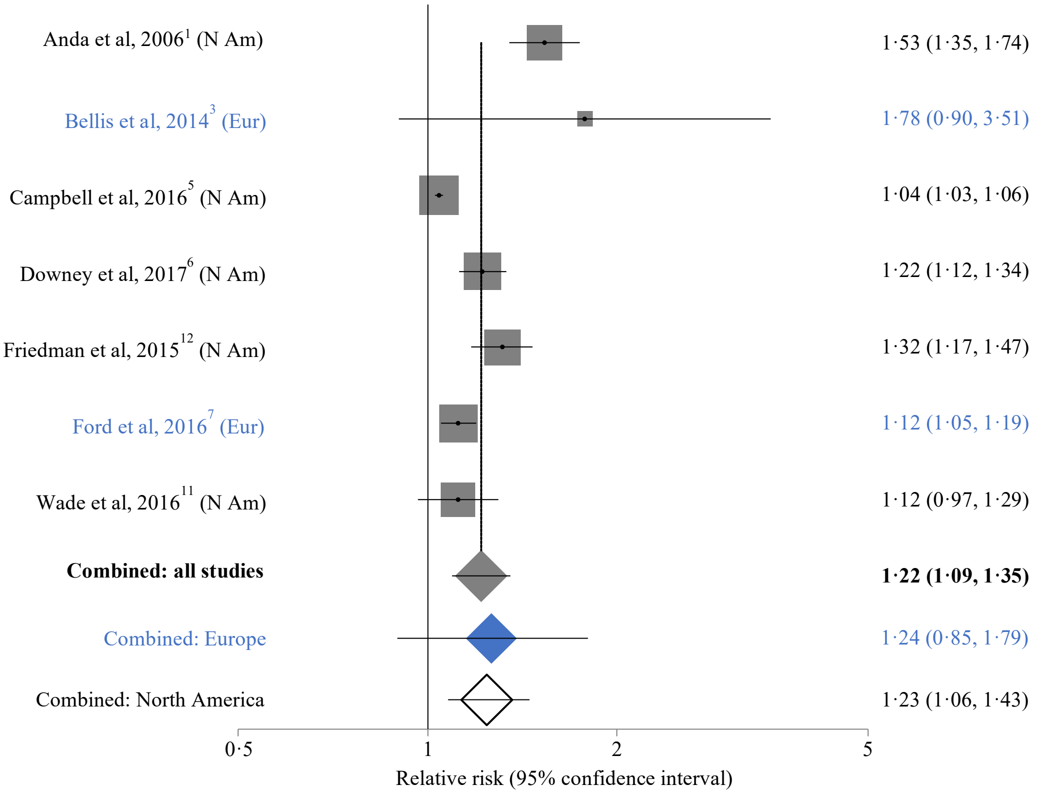

All studies: I^2 = 91·4% (95% CI = 85·3% to 94·3%); Cochran Q = 70·120336 (df = 6) P < 0·0001.

Europe (Eur): I^2 = 41·2% (95% CI = *% to *%); Cochran Q = 1·700557 (df = 1) P = 0·1922.

North America (N Am): I^2 = 93·8% (95% CI = 89·1% to 95·9%); Cochran Q = 64·796406 (df = 4) P < 0·0001.

Webfigure 8a: Increased risk of anxiety with 1 ACE: summary meta–analysis plot (random effects)

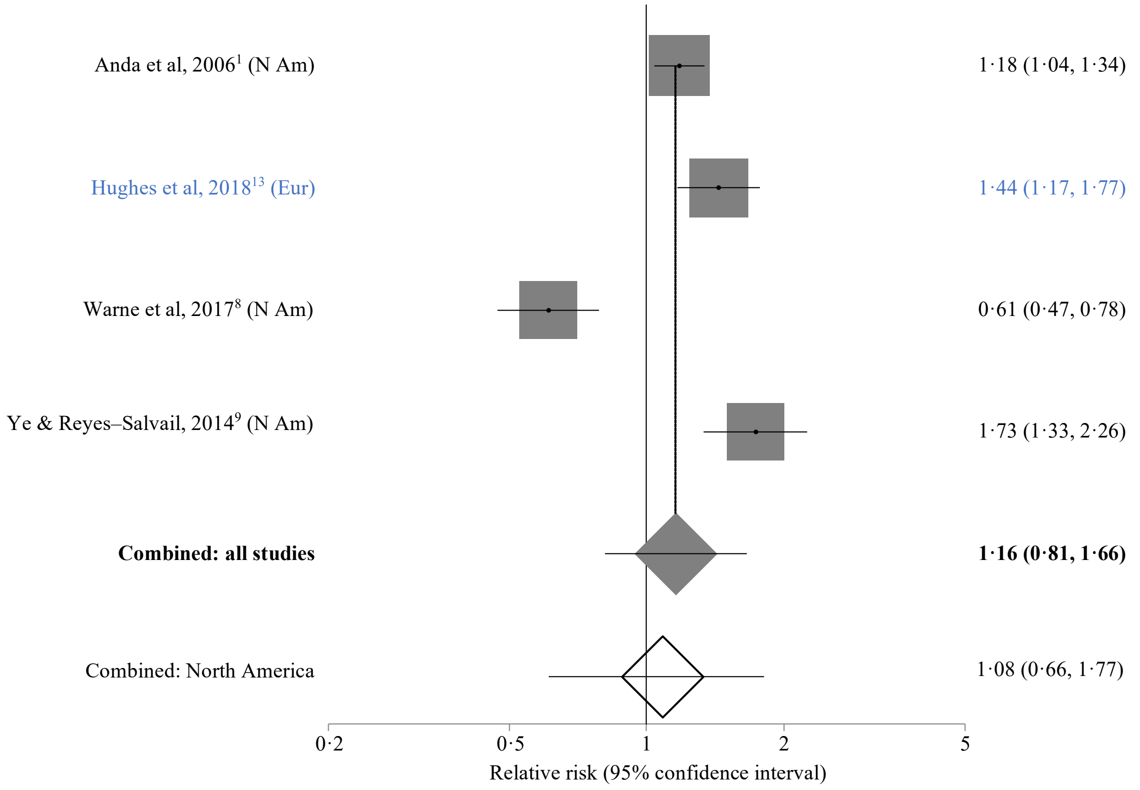

All studies: I^2 = 92% (95% CI = 82·4% to 95·3%); Cochran Q = 37·629662 (df = 3) P < 0·0001.
North America (N Am): I^2 = 94% (95% CI = 85·6% to 96·6%); Cochran Q = 33·115866 (df = 2) P < 0·0001.

Webfigure 8b: Increased risk of anxiety with ≥2 ACEs: summary meta–analysis plot (random effects)

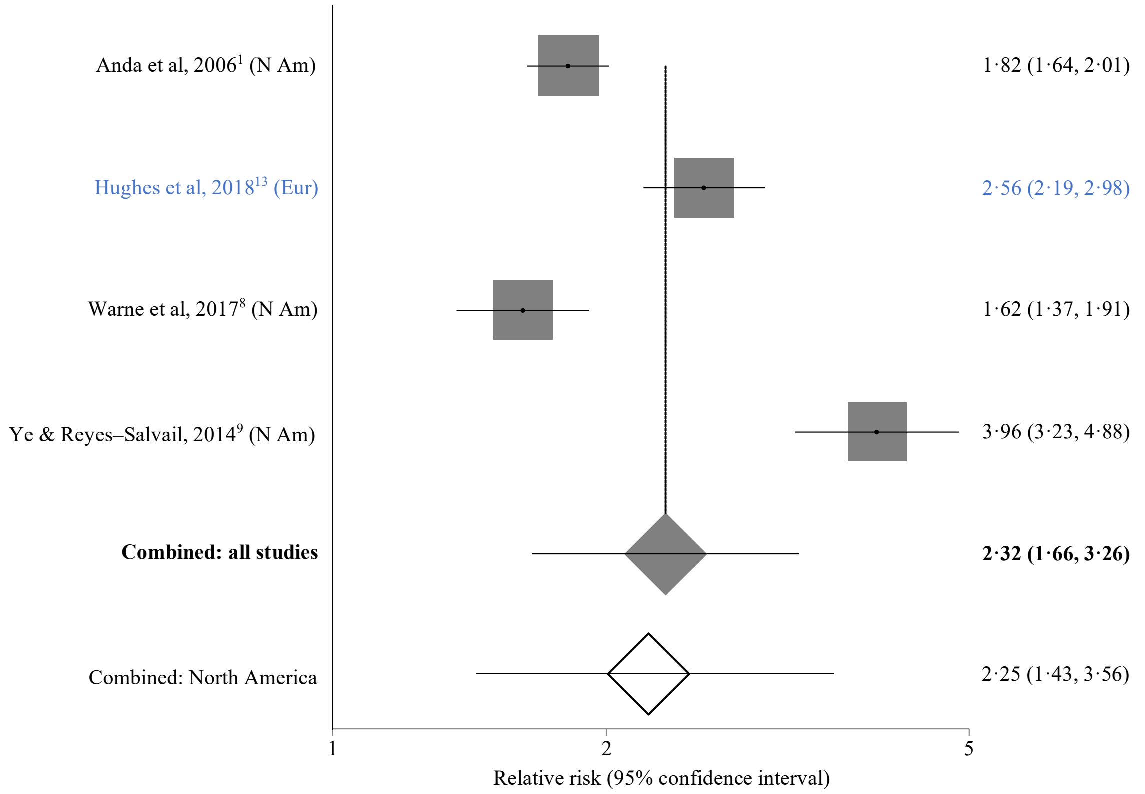

All studies: I^2 = 94·9% (95% CI = 90·7% to 96·7%); Cochran Q = 59·26137 (df = 3) P < 0·0001.
North America (N Am): I^2 = 96·1% (95% CI = 92·5% to 97·6%); Cochran Q = 51·610523 (df = 2) P < 0·0001.

Webfigure 9a: Increased risk of depression with 1 ACE: summary meta–analysis plot (random effects)

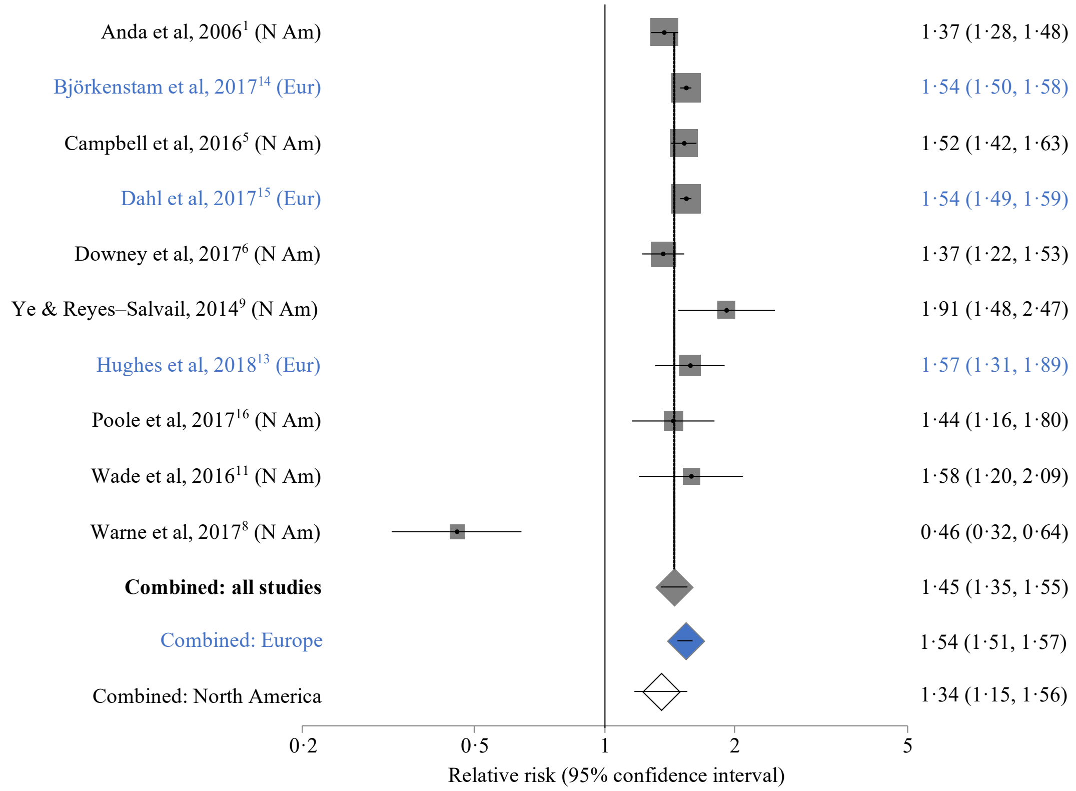

All studies: I^2 = 85·6% (95% CI = 74·6% to 90·5%); Cochran Q = 62·423067 (df = 9) P < 0·0001.
Europe (Eur): I^2 = 0% (95% CI = 0% to 72·9%); Cochran Q = 0·054135 (df = 2) P = 0·9733.
North America (N Am): I^2 = 88·6% (95% CI = 78·5% to 92·8%); Cochran Q = 52·582311 (df = 6) P < 0·0001.

Webfigure 9b: Increased risk of depression with ≥2 ACEs: summary meta–analysis plot (random effects)

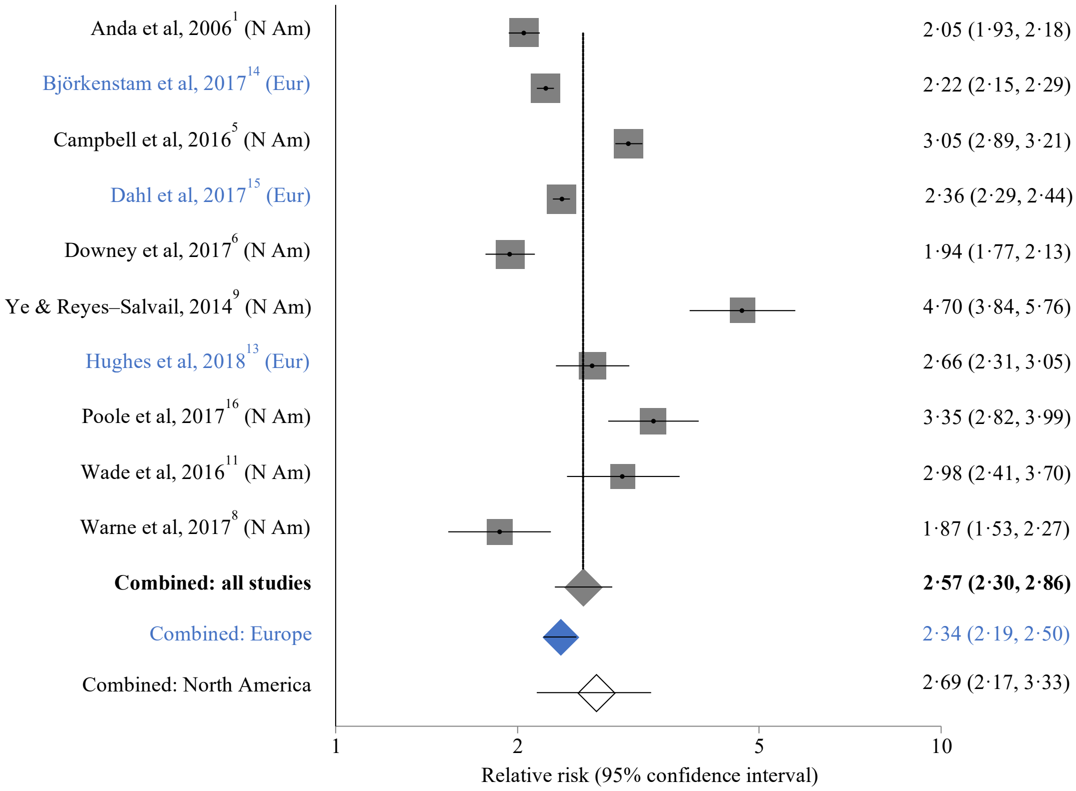

All studies: I^2 = 95·9% (95% CI = 94·6% to 96·8%); Cochran Q = 220·714334 (df = 9) P < 0·0001.
Europe (Eur): I^2 = 82·5% (95% CI = 0% to 92·5%); Cochran Q = 11·449759 (df = 2) P = 0·0033.
North America (N Am): I^2 = 96·8% (95% CI = 95·6% to 97·5%); Cochran Q = 186·817324 (df = 6) P < 0·0001.

Webfigure 9c: Increased risk of depression with 1 ACE: Funnel plot analysis

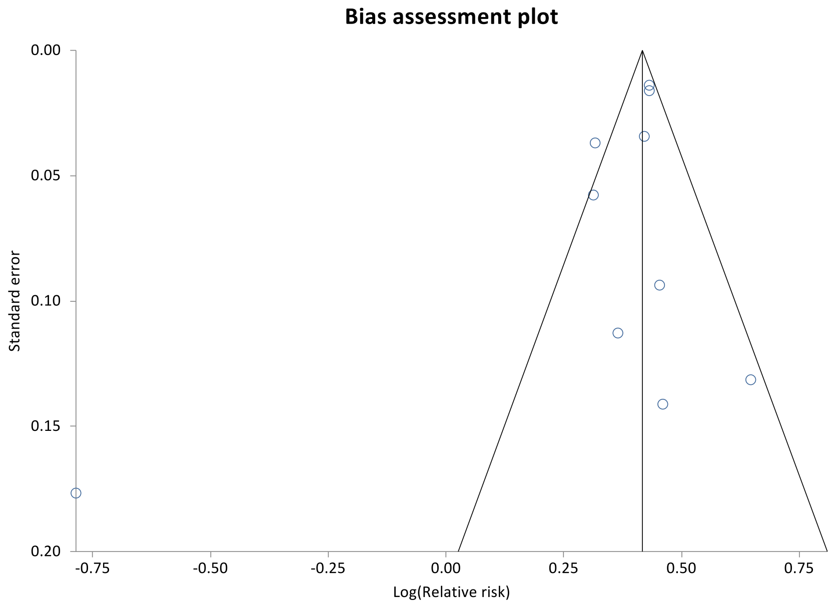

Begg–Mazumdar: Kendall's tau –0·244444, P = 0·2912; Egger: bias –1·631893 (95% CI = –4·341311 to 1·077524) P = 0·2023.

Webfigure 9d: Increased risk of depression with ≥2 ACEs: Funnel plot analysis

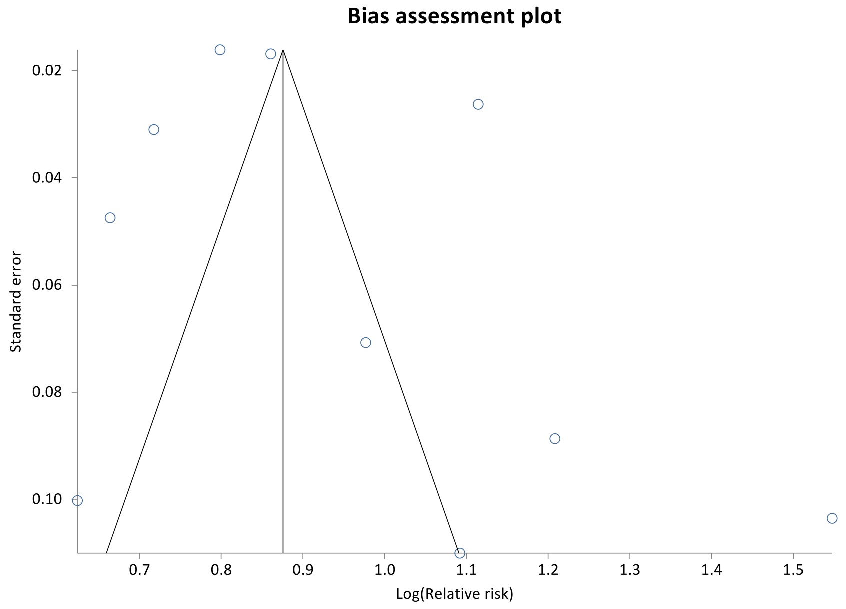

Begg–Mazumdar: Kendall's tau 0·377778, P = 0·1557; Egger: bias 2·456817 (95% CI = 3·701061 to 8·614696) P = 0·3845.

Webfigure 10a: Increased risk of cancer with 1 ACE: summary meta–analysis plot (random effects)

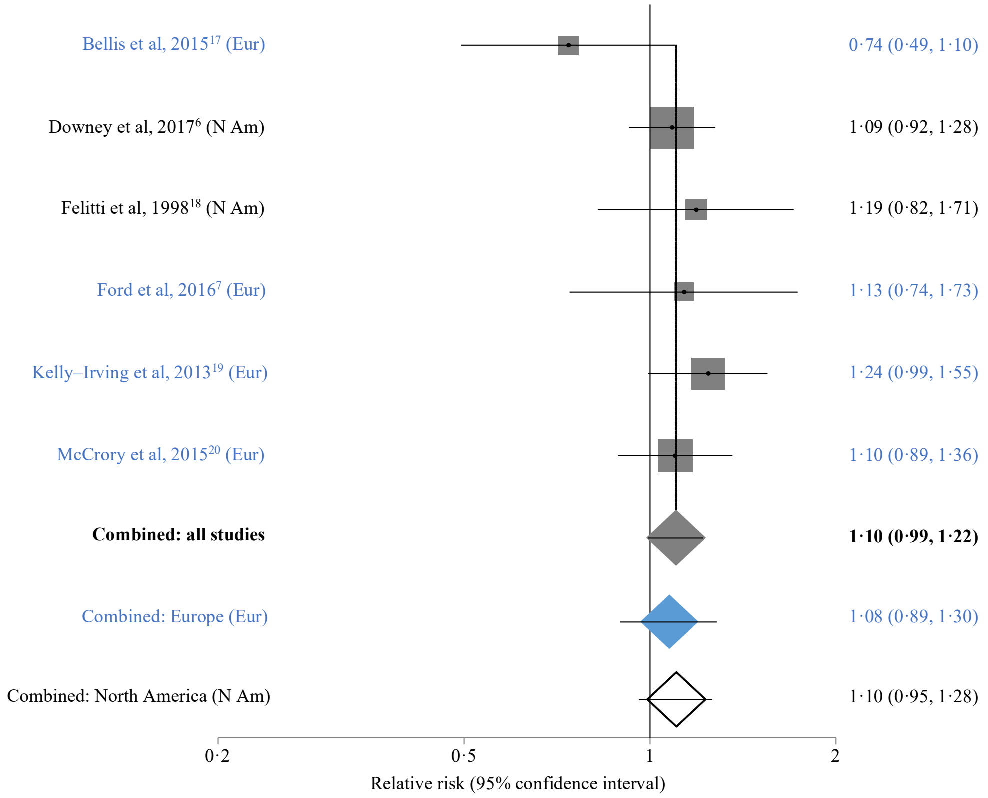

All studies: I^2 = 2·8% (95% CI = 0% to 62·1%); Cochran Q = 5·14498 (df = 5) P = 0·3984.
Europe (Eur): I^2 = 39·6% (95% CI = 0% to 79·1%); Cochran Q = 4·964743 (df = 3) P = 0·1744.
North America (N Am): I^2 = 0% (95% CI = *% to *%); Cochran Q = 0·188828 (df = 1) P = 0·6639.

Webfigure 10b: Increased risk of cancer with ≥2 ACEs: summary meta–analysis plot (random effects)

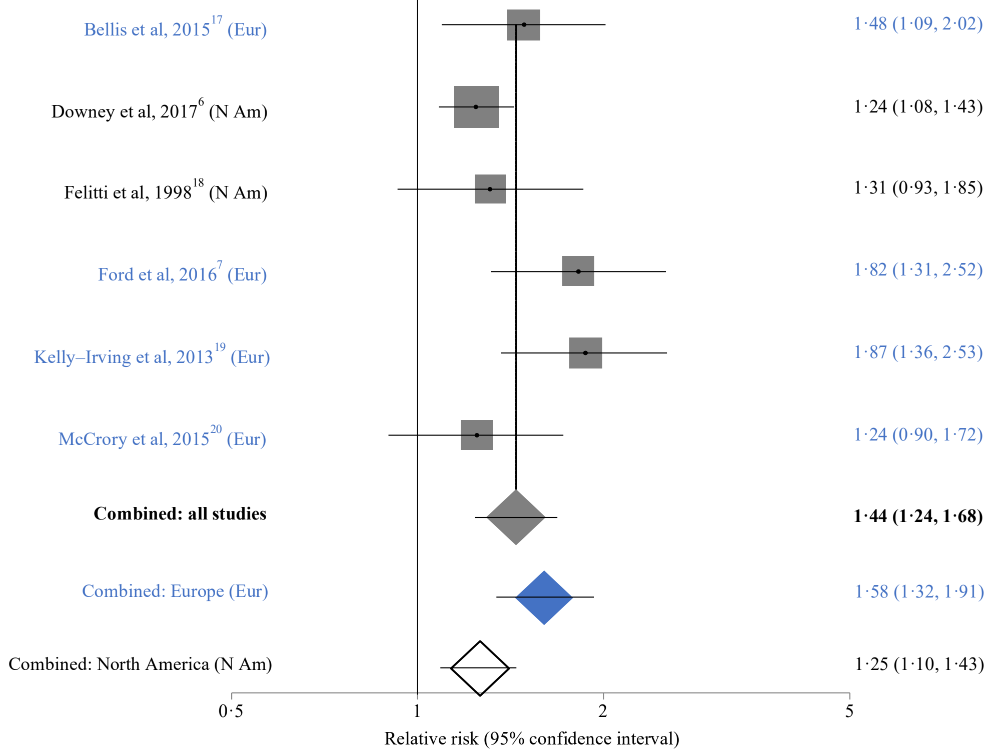

All studies: I^2 = 45·5% (95% CI = 0% to 76·8%); Cochran Q = 9·175265 (df = 5) P = 0·1023.
Europe (Eur): I^2 = 25·6% (95% CI = 0% to 75·4%); Cochran Q = 4·032113 (df = 3) P = 0·258.
North America (N Am): I^2 = 0% (95% CI = *% to *%); Cochran Q = 0·07578 (df = 1) P = 0·7831.

Webfigure 11a: Increased risk of cardiovascular disease with 1 ACE: summary meta–analysis plot (random effects)

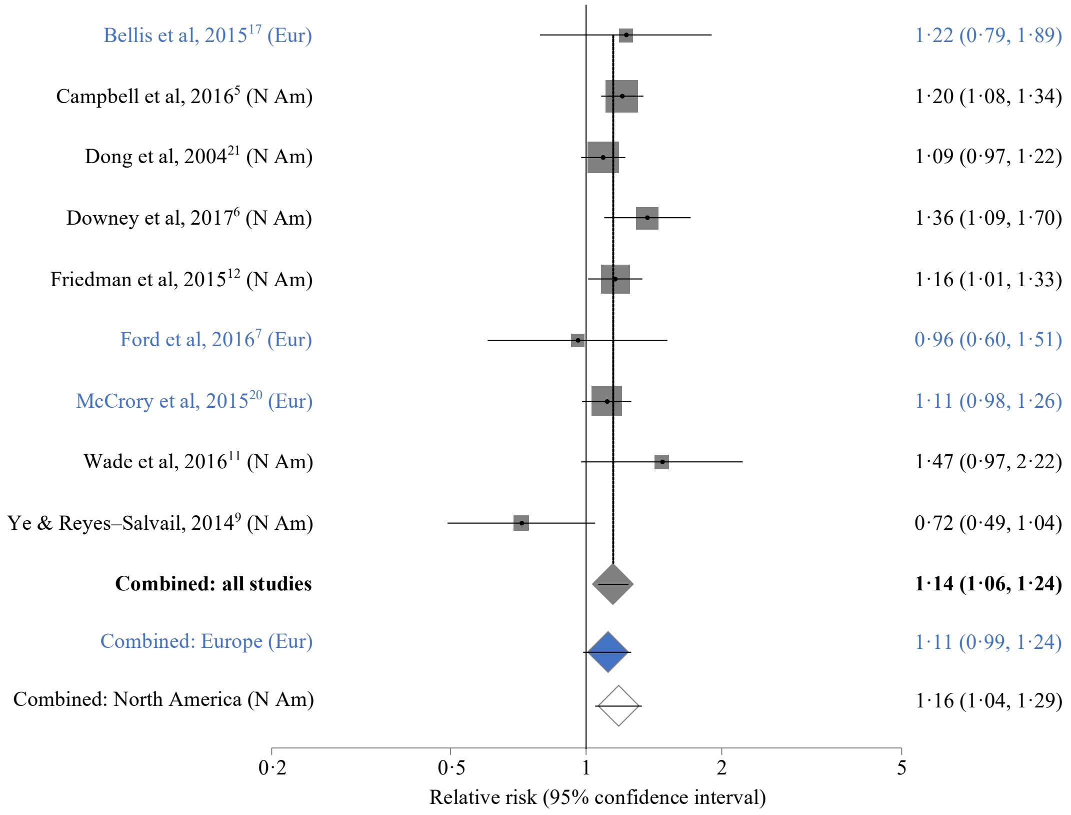

All studies: I^2 = 34·1% (95% CI = 0% to 68·6%); Cochran Q = 12·138907 (df = 8) P = 0·1451.
Europe (Eur): I^2 = 0% (95% CI = 0% to 72·9%); Cochran Q = 0·579104 (df = 2) P = 0·7486.
North America (N Am): I^2 = 55·2% (95% CI = 0% to 80%); Cochran Q = 11·151282 (df = 5) P = 0·0485.

Webfigure 11b: Increased risk of cardiovascular disease with ≥2 ACEs: summary meta–analysis plot (random effects)

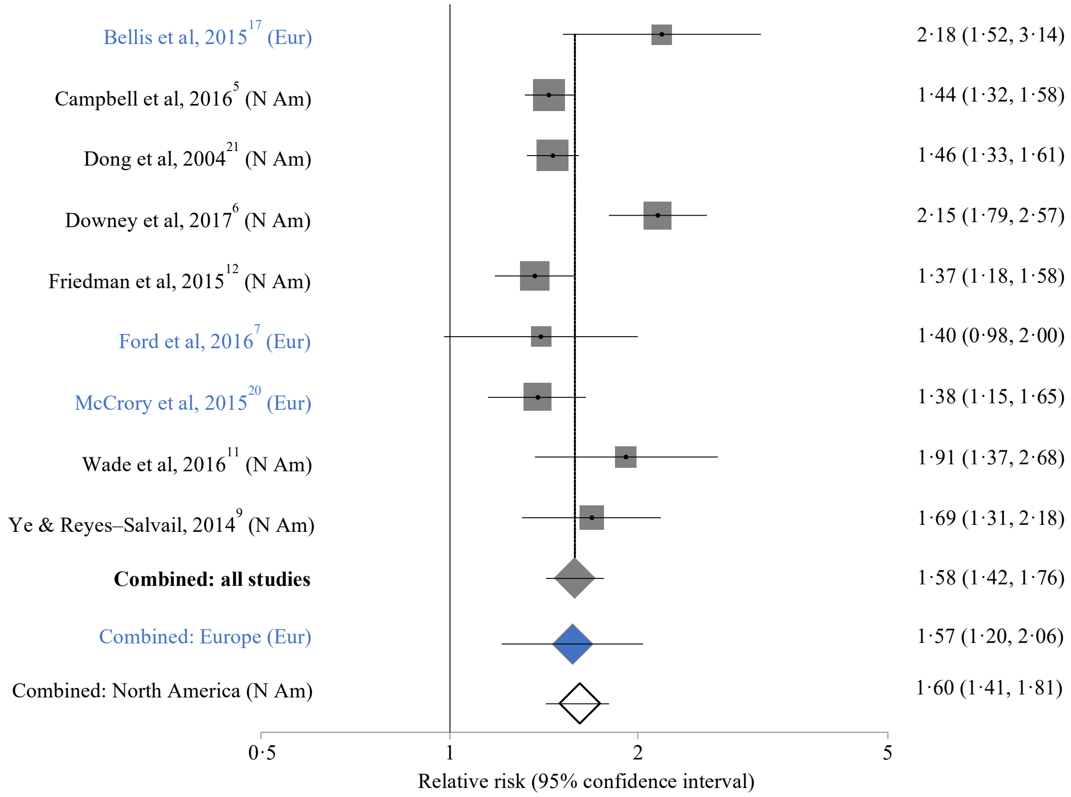

All studies: I^2 = 68·7% (95% CI = 21·8% to 82·7%); Cochran Q = 25·574243 (df = 8) P = 0·0012.
Europe (Eur): I^2 = 59·9% (95% CI = 0% to 86·7%); Cochran Q = 4·982537 (df = 2) P = 0·0828.
North America (N Am): I^2 = 75·7% (95% CI = 26·9% to 87·4%); Cochran Q = 20·571017 (df = 5) P = 0·001.

Webfigure 12a: Increased risk of diabetes with 1 ACE: summary meta–analysis plot (random effects)

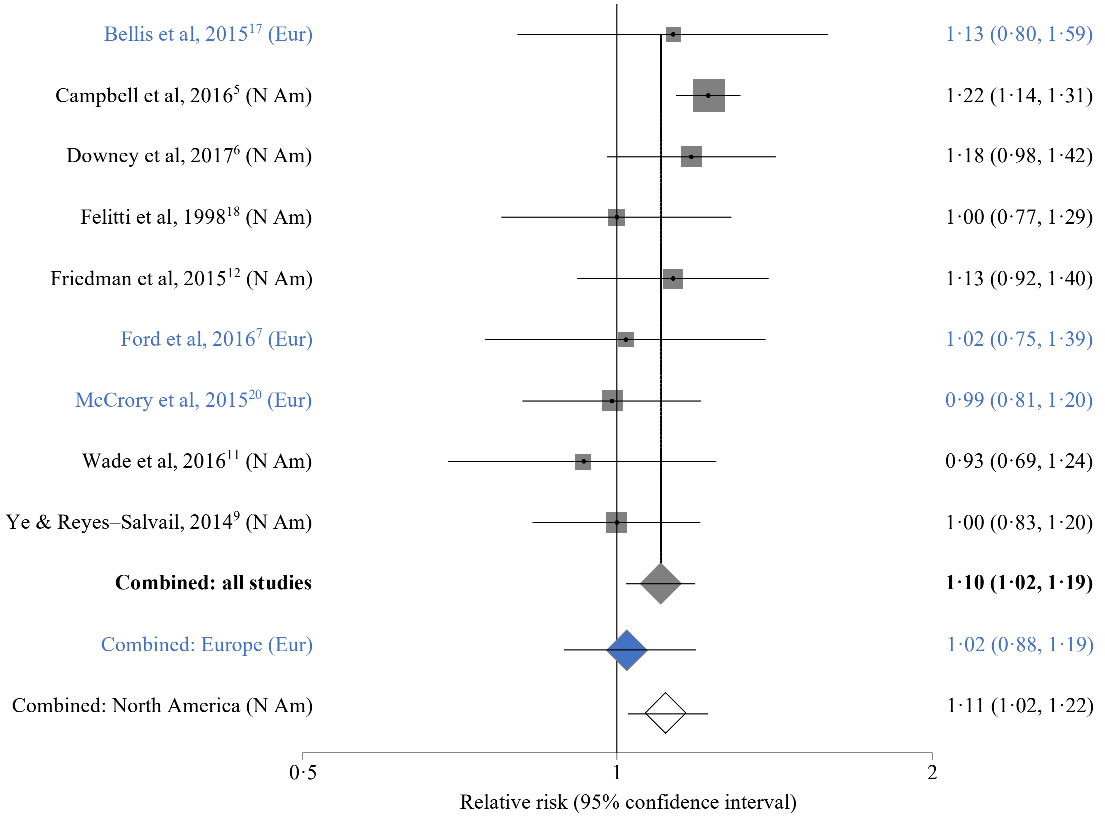

All studies: I^2 = 28·9% (95% CI = 0% to 66·5%); Cochran Q = 11·25188 (df = 8) P = 0·1878.
Europe (Eur): I^2 = 0% (95% CI = 0% to 72·9%); Cochran Q = 0·44664 (df = 2) P = 0·7999.
North America (N Am): I^2 = 39·3% (95% CI = 0% to 74·7%); Cochran Q = 8·23386 (df = 5) P = 0·1438.

Webfigure 12b: Increased risk of diabetes with ≥2 ACEs: summary meta–analysis plot (random effects)

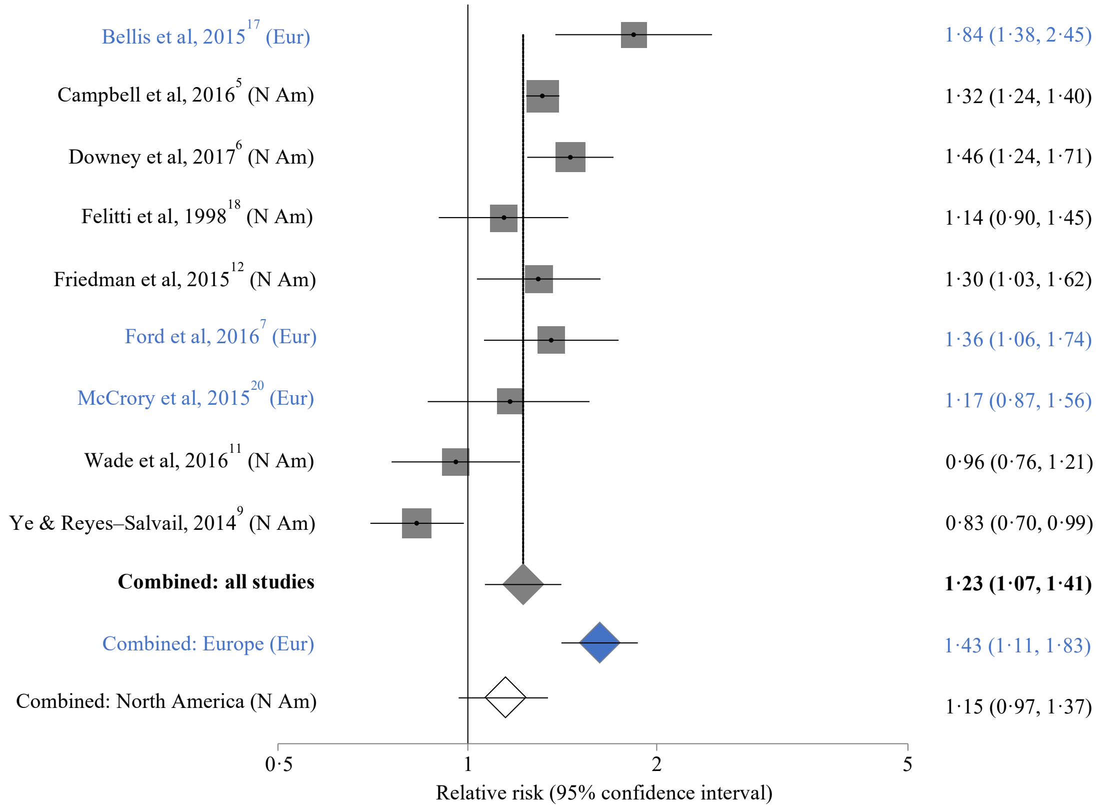

All studies: I^2 = 80·6% (95% CI = 60·3% to 88·2%); Cochran Q = 41·172328 (df = 8) P < 0·0001.
Europe (Eur): I^2 = 58·7% (95% CI = 0% to 86·4%); Cochran Q = 4·841264 (df = 2) P = 0·0889.
North America (N Am): I^2 = 85·3% (95% CI = 66·5% to 91·5%); Cochran Q = 33·958593 (df = 5) P < 0·0001.

Webfigure 13a: Increased risk of respiratory disease with 1 ACE: summary meta–analysis plot (random effects)

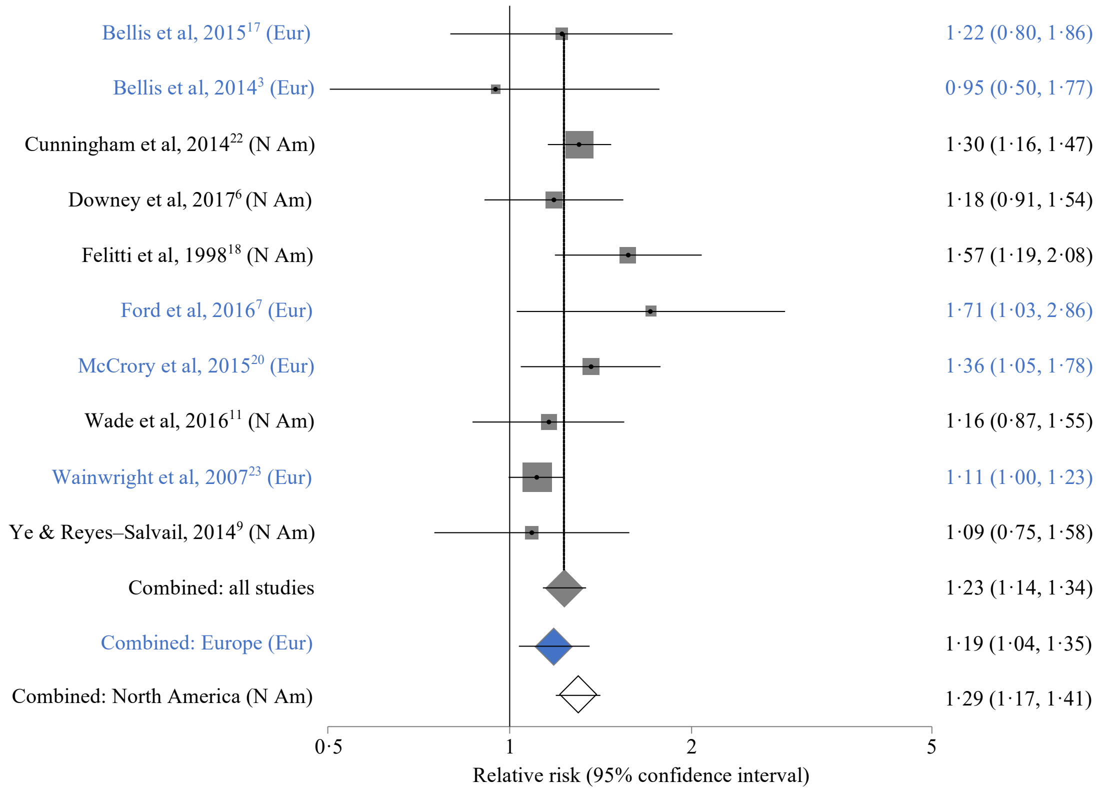

All studies: I^2 = 16·2% (95% CI = 0% to 60%); Cochran Q = 10·73484 (df = 9) P = 0·2943.
Europe (Eur): I^2 = 14·7% (95% CI = 0% to 69·1%); Cochran Q = 4·689212 (df = 4) P = 0·3207.
North America (N Am): I^2 = 0% (95% CI = 0% to 64·1%); Cochran Q = 3·607954 (df = 4) P = 0·4617.

Webfigure 13b: Increased risk of respiratory disease with ≥2 ACEs: summary meta–analysis plot (random effects)

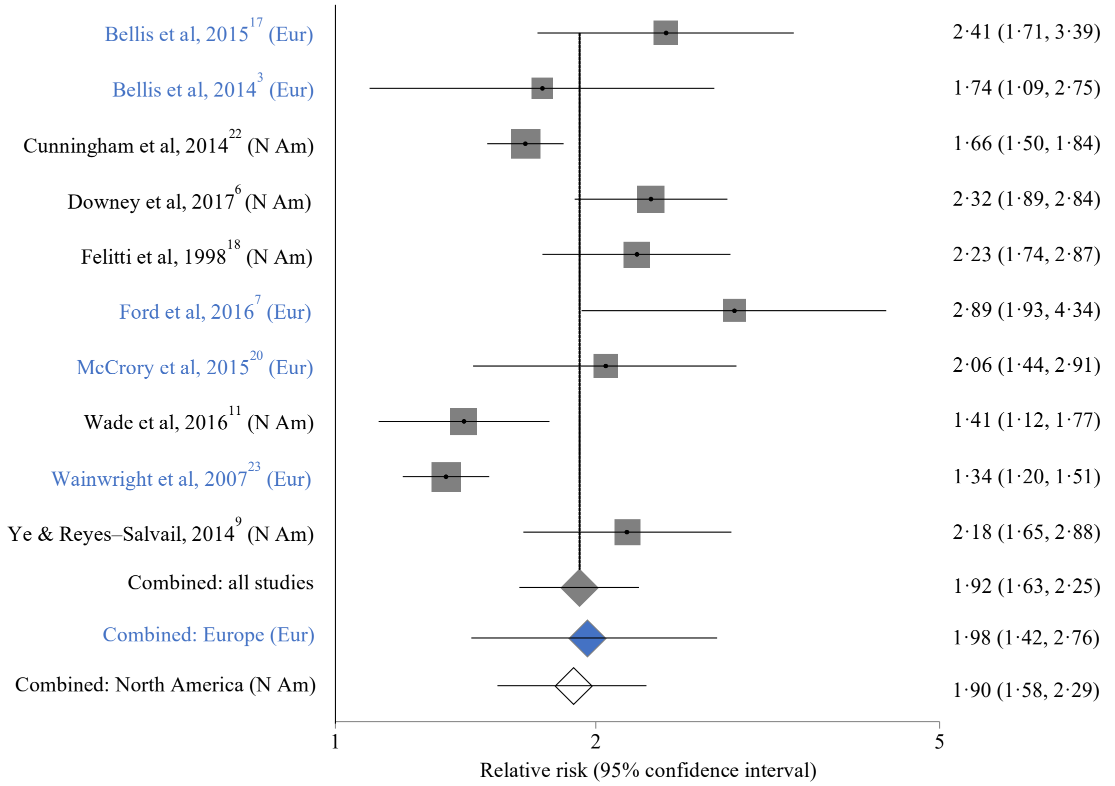

All studies: I^2 = 80·7% (95% CI = 62·7% to 88%); Cochran Q = 46·710121 (df = 9) P < 0·0001.
Europe (Eur): I^2 = 83·3% (95% CI = 53·1% to 91·1%); Cochran Q = 23·894287 (df = 4) P < 0·0001.
North America (N Am): I^2 = 77% (95% CI = 19·4% to 88·6%); Cochran Q = 17·359866 (df = 4) P = 0·0016.

Webfigure 13c: Increased risk of respiratory disease with 1 ACE: Funnel plot analysis

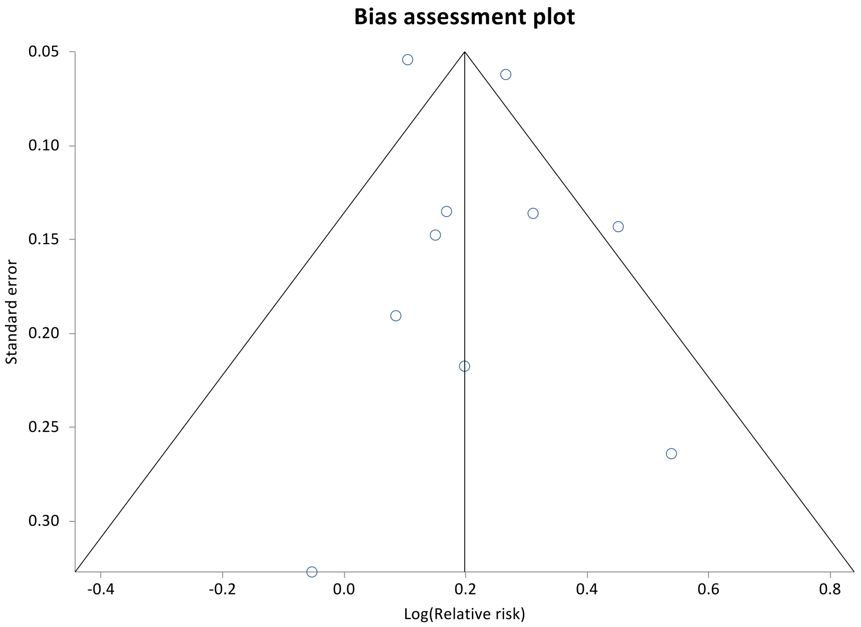

Begg–Mazumdar: Kendall's tau –0·066667, P = 0·7275; Egger: bias 0·49912 (95% CI = –1·064437 to 2·062678) P = 0·4827.

Webfigure 13d: Increased risk of respiratory disease with ≥2 ACEs: Funnel plot analysis

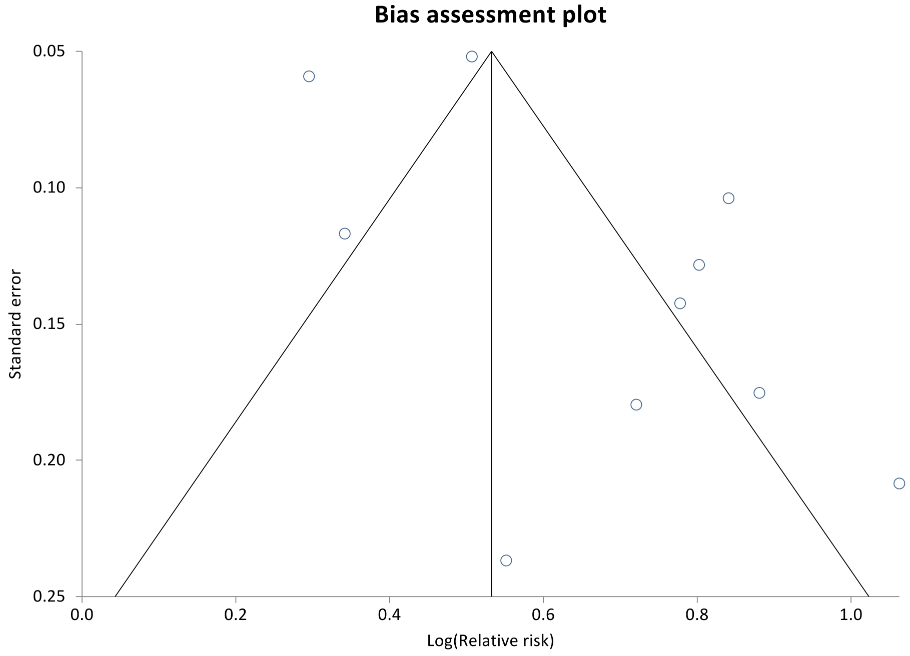

Begg–Mazumdar: Kendall's tau 0·155556, P = 0·6007; Egger: bias 0·935003 (95% CI = 0·129731 to 5·740274) P = 0·0423.

References

1. Anda RF, Felitti VJ, Bremner JD, et al. The enduring effects of abuse and related adverse experiences in childhood: A convergence of evidence from neurobiology and epidemiology. *Eur Arch Psychiatry Clin Neurosci* 2006; **256**: 174–86.
2. Bellis MA, Hughes K, Leckenby N, Perkins C, Lowey H. National household survey of adverse childhood experiences and their relationship with resilience to health–harming behaviors in England. *BMC Medicine* 2014; **12**: 72.
3. Bellis MA, Lowey H, Leckenby N, Hughes K, Harrison D. Adverse childhood experiences: retrospective study to determine their impact on adult health behaviours and health outcomes in a UK population. *J Public Health* 2014; **36**: 81–91.
4. Bellis MA, Hughes K, Leckenby N, et al. Adverse childhood experiences and associations with health–harming behaviours in young adults: surveys in eight eastern European countries. *Bull World Health Organ* 2014; **92**: 641–55.
5. Campbell JA, Walker RJ, Egede LE. Associations between adverse childhood experiences, high–risk behaviors, and morbidity in adulthood. *Am J Prev Med* 2016; **50**: 344–52.
6. Downey JC, Gudmunson CG, Pang YC, Lee K. Adverse childhood experiences affect health risk behaviors and chronic health of Iowans. *J Fam Viol* 2017; **32**: 557–64.
7. Ford K, Butler N, Hughes K, Quigg Z, Bellis MA. Adverse childhood experiences in Hertfordshire, Luton and Northamptonshire. Liverpool: Centre for Public Health, 2016.
8. Warne D, Dulacki K, Spurlock M, et al. Adverse childhood experiences (ACE) among American Indians in South Dakota and associations with mental health conditions, alcohol use and smoking. *J Health Care Poor Underserved* 2017; **28**: 1559–77.
9. Ye D, Reyes–Salvail F. Adverse childhood experiences among Hawai'i adults: findings from the 2010 Behavioral Risk Factor Survey. *Hawaii J Med Public Health* 2014; **73**: 181–90.
10. Ford ES, Anda RF, Edwards VJ, et al. Adverse childhood experiences and smoking in five states. *Prev Med* 2011; **53**: 188–93.
11. Wade Jr R, Cronholm PF, Fein JA, et al. Household and community–level adverse childhood experiences and adult health outcomes in a diverse urban population. *Child Abuse Negl* 2016; **52**: 135–45.
12. Friedman EM, Montez JK, Sheehan CM, Guenewald TL, Seeman TE. Childhood adversities and adult cardiometabolic health: Does the quantity, timing, and type of adversity matter? *J Aging Health* 2015; **27**: 1311–38.
13. Hughes K, Ford K, Davies AR, Homolova L, Bellis MA. Sources of resilience and their moderating relationships with harms from adverse childhood experiences. Wrexham: Public Health Wales, 2018.
14. Björkenstam E, Vinnerljung B, Hjern A. Impact of childhood adversities on depression in early adulthood: A longitudinal cohort study of 478,141 individuals in Sweden. *J Affect Disord* 2017; **223**: 95–100.
15. Dahl SK, Larsen JT, Petersen L, et al. Early adversity and risk for moderate to severe unipolar depressive disorder in adolescence and adulthood: A register–based study of 978,647 individuals. *J Affect Disord* 2017; **214**: 122–9.
16. Poole JC, Dobson KS, Pusch D. Childhood adversity and adult depression: the protective role of psychological resilience. *Child Abuse Negl* 2017; **64**: 89–100.
17. Bellis MA, Hughes K, Leckenby N, Hardcastle KA, Perkins C, Lowey H. Measuring mortality and the burden of adult disease associated with adverse childhood experiences in England: a national survey. *J Public Health* 2015; **37**: 445–54.
18. Felitti VJ, Anda RF, Nordenberg D, et al. Relationship of childhood abuse and household dysfunction to many of the leading causes of death in adults. The Adverse Childhood Experiences (ACE) Study. *Am J Prev Med* 1998; **14**: 245–58.
19. Kelly–Irving M, Lepage B, Dedieu D, et al. Childhood adversity as a risk for cancer: findings from the 1958 British birth cohort study. *BMC Public Health* 2013; **13**: 767.
20. McCrory C, Dooley C, Layte R, Kenny RA. The lasting legacy of childhood adversity for disease risk in later life. *Health Psychol* 2015; **34**: 687–96.
21. Dong M, Giles WH, Felitti VJ, et al. Insights into causal pathways for ischemic heart disease: adverse childhood experiences study. *Circulation* 2004; **110**: 1761–6.
22. Cunningham TJ, Ford ES, Croft JB, Merrick MT, Rolle IV, Giles WH. Sex–specific relationships between adverse childhood experiences and chronic obstructive pulmonary disease in five states. *Int J Chron Obstruct Pulmon Dis* 2014; **9**: 1033–43.
23. Wainwright NW, Surtees PG, Wareham NJ, Harrison BD. Psychosocial factors and asthma in a community sample of older adults. *J Psychosom Res* 2007; **62**: 357–61.
